# Leaf Thermal and Chemical Properties as Natural Drivers of Plant Flammability of Native and Exotic Tree Species of the Valparaíso Region, Chile

**DOI:** 10.3390/ijerph18137191

**Published:** 2021-07-05

**Authors:** Fabián Guerrero, Carla Hernández, Mario Toledo, Lorena Espinoza, Yulian Carrasco, Andrés Arriagada, Ariel Muñoz, Lautaro Taborga, Jan Bergmann, Camilo Carmona

**Affiliations:** 1 Department of Mechanical Engineering, Universidad Técnica Federico Santa María, Avenida España 1680, Valparaíso 2390123, Chile; carla.hernandez@alumnos.usm.cl (C.H.); mario.toledo@usm.cl (M.T.); lorena.espinoza.11@alumnos.usm.cl (L.E.); andres.arriagada@usm.cl (A.A.); ccarmona.young@gmail.com (C.C.); 2 Forestry Department, Faculty of Forestry and Agricultural Sciences, Universidad de Pinar del Río, Calle Martí 300, Pinar del Río CP 20100, Cuba; yulianc84@gmail.com; 3 Institute of Geography, Pontificia Universidad Católica de Valparaíso, Avenida Brasil 2241, Valparaíso 2362807, Chile; ariel.munoz@pucv.cl; 4 Centro de Acción Climática, Pontificia Universidad Católica de Valparaíso, Avenida Brasil 2950, Valparaíso 2340025, Chile; 5 Centro de Ciencia del Clima y la Resiliencia (CR)2, Santiago 8320000, Chile; 6 Natural Products Laboratory, Department of Chemistry, Universidad Técnica Federico Santa María, Avenida España 1680, Valparaíso 2390123, Chile; lautaro.taborga@usm.cl; 7 Institute of Chemistry, Science Faculty, Pontificia Universidad Católica de Valparaíso, Avenida Universidad 330, Valparaíso 2373223, Chile; jan.bergmann@pucv.cl

**Keywords:** flammability, fire behavior, forest fire, sclerophyllous species, organic metabolites

## Abstract

Forest fires are one of the main environmental threats in Chile. Fires in this Mediterranean climate region frequently affect native forests and exotic plantations, including in several cases urban and rural settlements. Considering the scarcity of information regarding the fire response dynamics of tree species that are frequently affected by fires, this study aims to establish a flammability classification according to the evolution of the fire initiation risk presented by the most affected forest species in the Valparaíso region. Three exotic species, *Eucalyptus globulus*, *Pinus radiata*, and *Acacia dealbata*, and two native species, *Cryptocarya alba* and *Quillaja saponaria*, were studied. Flammability assays indicate that *E. globulus*, *A. dealbata*, and *C. alba* are extremely flammable, whereas *P. radiata* and *Q. saponaria* are flammable. Furthermore, *E. globulus* and *A. dealbata* have the highest heating values while *Q. saponaria* has the lowest values. The extreme flammability of *E. globulus, A. dealbata*, and *C. alba* indicates a high susceptibility to ignite. Furthermore, the high heat of combustion of *E. globulus* and *A. dealbata* can be associated with a high energy release, increasing the risk of fires spreading. In contrast, *Q. saponaria* has the lowest predisposition to ignite and capacity to release heat. Accordingly, this work shows that all studied tree species contain organic metabolites that are potentially flammable (sesquiterpenes, aliphatic hydrocarbons, alcohol esters, ketones, diterpenes, and triterpenes) and can be considered as drivers of flammability in vegetation. Finally, these preliminary results will aid in the construction of more resilient landscapes in the near future.

## 1. Introduction

Forest fires are one of the most frequent and important disturbances and environmental threats to forest ecosystems at both a local and global scale [1]. Over the last few decades, wildfires have deeply affected human lives and public infrastructure, risking public health by air pollution from wildfire smoke, a complex mixture of pollutants which impacts communities due to the emission of particulate matter (PM_10_, PM_2.5_ and PM_1_), carbon dioxide (CO_2_), carbon monoxide (CO), methane (CH_4_), sulphur dioxide (SO_2_), and polycyclic aromatic hydrocarbons (PAHs), among others [2,3,4,5]. Among the effects of forest fire emissions are contributions to the greenhouse effect, local atmospheric pollution events, increased cardiorespiratory afflictions and increased amounts of aerosols that are capable of producing significant damages to ecosystems and a population’s health [6,7,8,9]. In addition to the physical impacts, there are psychological and psychiatric impacts associated with these disasters [5], such as post-traumatic stress [10] or identity and emotional problems resulting from drastic changes to the landscape and the destruction of livelihoods [11].

Moreover, there has been an increase in the number of wildland fires and affected areas, primarily in Mediterranean regions [12,13,14]. For example, in 2017, the number of forest fires in Europe increased by almost 200% compared to the average of the last decade, with Spain, Portugal, Greece, Italy, and France regularly affected by disasters due to fire [14]. In addition to Mediterranean areas, the effects of forest fires have increased in other regions of the world. For example, in Alaska, during the last decade, 2.5-times more surface was burnt compared to the previous decade. In 2019 alone, a total of 378 fires were registered, affecting 2,525,356 acres [15]. In addition, California registered one of its most destructive and deadly fires during the period of 2018–2019, affecting more than 1.2 million hectares [16,17]. In Australia, more than 27 million hectares were affected in September 2019, with the start of the fire season being much earlier compared to previous years [18]. Although more than 90% of forest fires worldwide are related to anthropogenic causes, climatic factors have intensified the spread and destructive power of wildfire events [19,20,21]. These climatic factors correspond primarily to high summer temperatures and reduced levels of precipitation, resulting in long drought periods that decrease the water content of plants [20,21,22].

Chile is home to approximately 136,000 km^2^ of native forest and 22,000 km^2^ of forest plantations. As wildland fires are a common threat during the summer season, there is a need to address and investigate this environmental challenge. The regions most affected by forest fires are located in the central and southern regions of the country, which have a Mediterranean climate characterized by periods of intense rain during the winter and very dry summers [19]. The largest and most damaging forest fires in the history of Chile took place in the period 2017–2018. In total, around 5760 km^2^ of forest area was burned between the Valparaíso and Araucanía regions, which was five times greater than the period with the previous largest affected area (2014–2015). During the season of 2017–2018, forestry plantations (223,650 ha), native forests (60,995 ha), native shrublands (187,906 ha) and rural properties [20,21,23] were affected. In three months, more than 1157.1 km^2^ of native sclerophyllous forests and forest plantations were burned, with more than 4000 people engaged in combating fire [23] and more than 9.5 million people exposed to high concentrations of particulate matter, causing an estimated 76 premature deaths and 209 additional hospital admissions for respiratory and cardiovascular conditions [24].

Valparaíso, the region of the country with the third highest occurrence of wildfires between 1977 and 2020 [23], was also affected, including native and endemic forest species such as *Cryptocarya alba, Quillaja saponaria*, and *Lithraea caustica* (25,402 km^2^ burnt), as well as forestry plantations of *Eucalyptus globulus* (6448 km^2^ burnt) and *Pinus radiata* (0.283 km^2^ burnt) [23]. Based on these data, native vegetation was most affected in terms of burnt surface, primarily through the degradation of sclerophyllous forest ecosystems [25]. Moreover, in the Valparaíso region, a wildfire called “La Engorda—Peñuelas Lake National Reserve (PLNR)” affected the cities of Valparaíso and Quilpué for eight consecutive days (January 14 to 21, 2021). This disaster consumed an area of 3900 ha of grassland, scrub, eucalyptus and native forest species. Due to the rapid spread of flames, the National Emergency Office (ONEMI) requested the preventive evacuation of the habitants of different residential sectors of Quilpué, including Los Pinos, Colinas de Oro, and Teniente Serrano, among others [26].

It should be noted that the most influential climatic factors include, in addition to the increase in temperature and the occurrence of heat waves, the fact that the reduction in the level of precipitation generates drought conditions that are directly related to the frequency and intensity of fire events [27]. As a matter of fact, Chile has seen an increase in the occurrence of fire events after the longest and intensive drought recorded during the last millennium, with an average rainfall deficit of 20–40% since 2010 [28]. This prolonged drought caused hydric stress in vegetation, thereby resulting in a large amount of highly flammable material that is susceptible to ignition [29,30].

At the same time, an increase in the occurrence and severity of fire events is projected based on the prediction of adverse weather conditions. Specifically, projections of climate variability in Chile estimate a decrease of up to 15% in precipitation levels and an increase in temperatures of between 0.5 and 1.5 °C towards 2030 and more than 2.0 °C by the end of the 21st Century, with a 40% reduction in precipitation and a 4 °C increase in average temperatures [31,32,33]. This, combined with population and urban growth, results in a higher level of exposure to socio-environmental disasters, generating the need to develop strategies and tools to reduce the vulnerability of the population to the threat of fire, thus improving the adaptive capacity and resilience of communities while conserving the ecosystem services of the Mediterranean forest in the context of sustained climate change.

In this context, it is crucial to study the natural factors that influence the flammability of vegetation, particularly those intrinsic factors that act as fire drivers, in order to develop an effective planning and management approach for territories vulnerable to fire events, such as those in the wildland–urban interface (WUI), rural sectors, and conservation areas.

The most commonly used definition for the flammability of forest species was described by Anderson in 1970 [34] based on the following three components: ignitability, sustainability, and combustibility. Subsequently, Martin et al. [35] considered a fourth phenomenon called consumability [36]. Furthermore, a flammability index (FI) was proposed by Valette in 1990 [37] for Mediterranean forest species, which considers ignition time, flame duration, and burning time. This index is used in different parts of the world as a simple technique for evaluating flammability parameters and is still widely used by various authors [38,39,40,41,42,43,44,45,46,47] to study and classify tree species according to their flammability in order to find those that are less flammable and useful for forest fire risk management. Simultaneously, there is a need to understand the relationship between forest fuels and their thermochemical parameters, including heating values, to be able to include them in wildfire risk evaluations [48,49,50]. Pausas et al. [50] suggested that measures of flammability should include a number of forest fuel characteristics that influence the probability of ignition and fire behavior and encompass three main components: ignitability, heat release, and fire spread rate. In addition to climatic factors that influence the development of fire, the authors suggest that the three components of flammability can explain the ecological impact generated by forest fires. For example, heat release is often related to latent heat, which increases soil temperature and influences the probability of fire-related damage [50]. Furthermore, the heating value of a fuel can be associated with the rate at which a fire spreads, as it represents the energy transmitted along the fuel material or to neighboring tree species in a forest context [51,52]. However, in addition to the heating value, which is related to the composition and structure of the fuel, other factors such as humidity, oxygen, and environmental conditions also influence the spread of fire [51]. Moreover, internal factors of vegetation, such as physical (leaf thickness, surface area, and perimeter, among others) and chemical properties (lignin content, moisture content, mineral content, and volatile content) of leaves and leaf litter are wildfire drivers through their effect on flammability [53]. Among these, the chemical composition is one of the most important natural factors that influences the flammability of plants, particularly terpene content (monoterpenes and sesquiterpenes), where it has been postulated that the initiation and spread of forest fires is directly influenced by the essential oils/resins contained in woody species, due to the accumulated terpenes within this type of tree [54]. There is scientific evidence that terpenes increase the risk of forest fires. Volatile terpenes (monoterpenes and sesquiterpenes) have a high degree of flammability due to their high calorific values, low flash points, and low flammability limits, increasing the flammability of vegetation [38,42,55,56,57].

The effort to associate natural factors with the flammability of vegetation has allowed an understanding of fire ecology and for researchers to develop tools and methodologies for forest management planning that can range from non-spatial conceptual models to techniques that include linear programming, binary search, cost–benefit analysis, simulation, and heuristics, among others [58]. Recently, Molina et al., 2017 [59] proposed a methodology based on the risk evaluation for forest fires in wildland–urban interfaces (WUI) in Mediterranean areas. Through this model, an ignition index that indicated a fuel’s availability to ignite and spread through tree species was developed. This index is calculated based on the probability of ignition (meteorological conditions), the ignition coefficient (fuel characteristics), and flammability. Furthermore, different ways to determine the behavior of forest fires have been studied through simulation models [59,60,61,62,63], where the fire parameters (linear propagation velocity, first-line intensity, and flame length) obtained by Castillo et al., 2020 [63] were comparable with the areas under study. The behavior of fire (intensity, gravity) can be obtained from environmental features, climate change, and vegetation characteristics, such as moisture content, heating value, and the potential for fire to spread. However, flammability parameters that could possibly improve efficiency in order to better understand the dynamics of vegetation responses in a forest fire have not yet been considered. Moreover, Castillo et al., 2020 [63] emphasize that the importance of diversity in the physical properties of native and exotic forest species has not yet been addressed. Based on the results, high severity and damage as a result of forest fires are predicted, principally in areas with higher amounts of dry biomass.

The National Forest Corporation of Chile (CONAF) uses an ignition probability index as a wildfire prevention strategy, based on a matrix of meteorological data that includes solar radiation and temperature, as well as the humidity of the forest fuel, based on geospatial data (at a global scale) [64]. However, experimental data on the thermochemical properties (flammability, heating values), moisture, and terpene content of both native and exotic forests in Chile are scarce, highlighting the need to research these properties to expand and deepen our existing knowledge and to improve fire hazards, as well as to develop short- and long-term fire prevention and management strategies.

In Chile, little is known about the flammability of different tree species. This issue has been an intense topic in the discussion between academics, foresters, and the forest industry. However, there is no information about the flammability of the main exotic and native species of trees covering the Chilean landscapes, especially in the south-central zone, where most of the population settlements and the forest industry are located.

In this context, the objective of this research was to develop a study of the thermochemical properties (flammability, heating value, and flash point) of five of the most affected sclerophyllous tree species by wildfires in the Valparaíso region [23] and to evaluate the relationship between these properties with the flammability drivers (organic metabolites and moisture content) present in the vegetation. This information could contribute to the improvement of fire models and fire forecasting as the flammability of leaves in relation to their chemical composition is highly relevant, due to the inadequate knowledge available in the subject. This research would help to create fire management tools, such as a fire risk classification method for native species and the use of low-flammability vegetation as a natural fence for specific areas and/or plantations, and to develop mitigation strategies, as well as for the management of forest fires in order to reduce the impact of this type of event.

## 2. Materials and Methods

### 2.1. Study Area and Leaf Sampling

**Figure 1:**
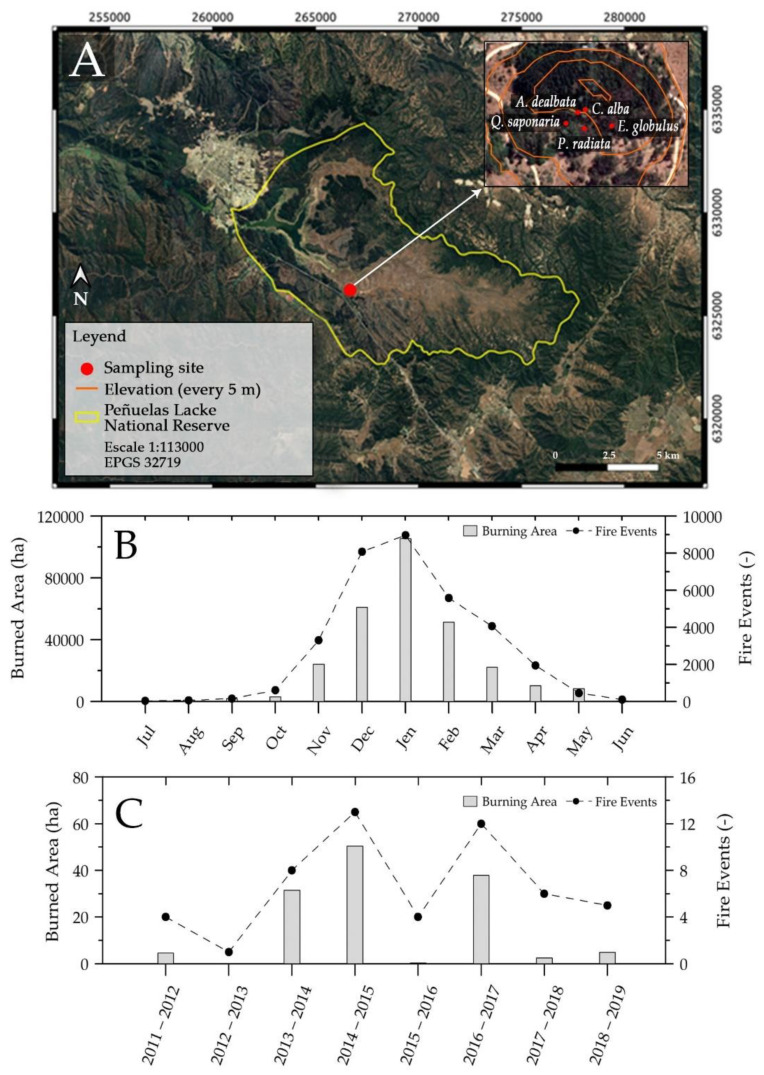
Peñuelas Lake National Reserve (PLNR): (**A**) geographic location, sampling site, and detail of species under study, (**B**) monthly sum of historical burned area and fire events from 1985 to 2020 in Valparaíso Region [23], and (**C**) fire regime reported by the administration of PLNR for the summer seasons of 2011–2012 to 2018–2019.

The sample collection was carried out in the Peñuelas Lake National Reserve (PLNR) during the summer of 2018–2019. The reserve location, sampling site, forest species under study, fire regime on a regional scale basis from historical data (from 1985 to 2020), and fire activity within the national reserve during the summer season (2011–2012 to 2018–2019) are depicted in Figure 1.

Reports of regional-scale forest fire activity based on historical data (1985 to 2020, see Figure 1B) indicate that more than 80% of wildfire events in the Valparaíso region occur in summer periods [23], where 96% of these events are concentrated in the period November–April. Accordingly, the highest amount of burnt surface is concentrated in the summer period, which shows a consistency over time regarding historical data.

**Figure 2:**
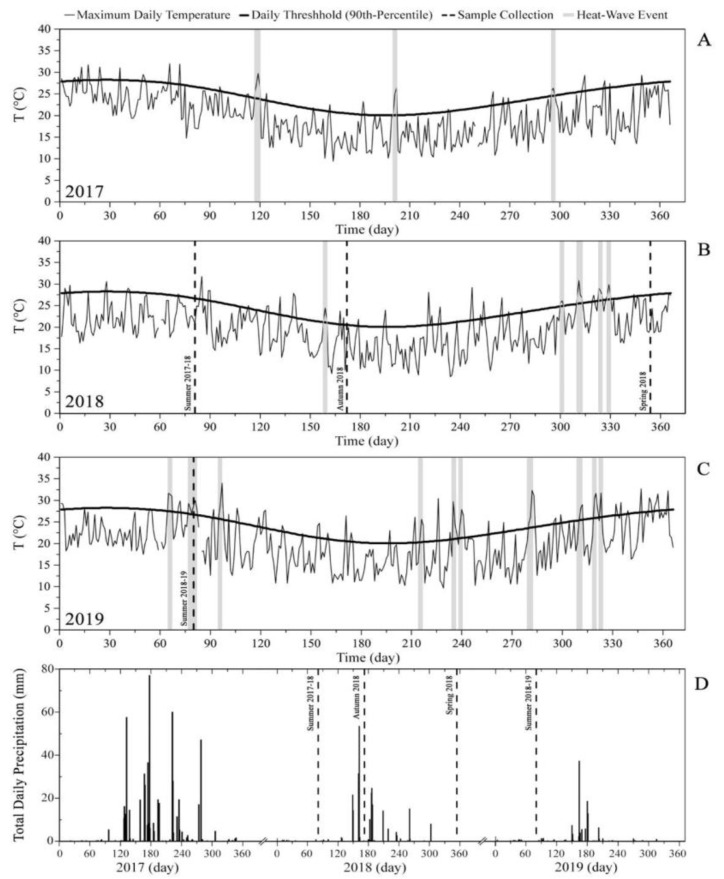
Extreme climatic conditions during the period 2017–2019: evolution of maximum daily temperature (**A**–**C**) and daily total precipitation level (**D**) according to the database from Rodelillo Station (National code 330007, Directorate Meteorological of Chile). Gray areas represent the occurrence of heatwaves during the period under study.

The geo-climatic characteristics and distribution of plant species, in addition to the impact of fire in the area, have been described by Guerrero et al., 2020 [6] and Hauenstein et al. [65]. During 2018, the study area registered an average annual temperature of 13.4 °C and a total accumulated precipitation of 257.1 mm, according to the database of the Rodelillo Meteorological Station, located 13 km north of the sampling location (National code 330007, Directorate Meteorological of Chile) [66]. Extreme meteorological conditions were registered in the Valparaíso region between 2017 and 2019. Figure 2 presents data from Rodelillo Station corresponding to daily maximum temperatures and thresholds (90th percentile) of heat waves in Chile during 2017 (Figure 2A), 2018 (Figure 2B), and 2019 (Figure 2C). Figure 2D presents the daily evolution of accumulated precipitation between 2017 and 2019. Regarding the daily maximum temperature, a sustained increase in the occurrence of heat wave events was observed, registering 3, 5, and 10 events during 2017, 2018, and 2019, respectively. The latter year also saw reduced precipitation levels.

Five woody species were sampled, comprising two native species (*Q. saponaria* and *C. alba*) and three exotic species (*E. globulus*, *P. radiata*, and *A. dealbata*). These species were selected as they are representative in the sclerophyllous forest ecosystem and the most affected forest species by wildfires in the area. In addition, these species are the most abundant in the Valparaíso region and also in the sampling site of this research (PLNR), with 71% *E. globulus*, 21% *P. radiata*, 3% *A. dealbata*, 1% *Q. saponaria*, and 1% *C. alba* in the area [6].

Fully expanded, healthy leaves were collected from the outer exposed plant canopy, using the procedure described by Guerrero et al., 2020 [6] in terms of sun exposure.

The variation in the flammability of live foliage is critical given that leaves are one of the first structures to ignite [67] and contribute a large amount of fuel for forest fires [68]. Leaves have significant importance in spreading fire at the landscape scale [69]. Moreover, the fluctuation of leaf flammability among forest species provides opportunities for wildland fires to expand horizontally within a plant stratum and also vertically to the upper canopy [70].

For leaf collection, mixed canopy sampling was developed to capture intraspecific differences derived from leaf exposure to sun and shade. In particular, plants have evolved a variety of morpho-physiological and biochemical adaptations that optimize the interception, absorption, and processing of light to which leaves are exposed. In this case, sun-exposed leaves are small and thick, with well-developed palisade tissue and higher stomatal density, while shaded leaves maximize light capture but lower the maintenance costs of excess photosynthetic machinery, producing thinner, lighter leaves with a larger specific area. Furthermore, shaded leaves show evidence of higher chlorophyll concentrations, higher ATPase activity, and lower Rubisco content compared to sun leaves [71,72].

**Figure 3:**
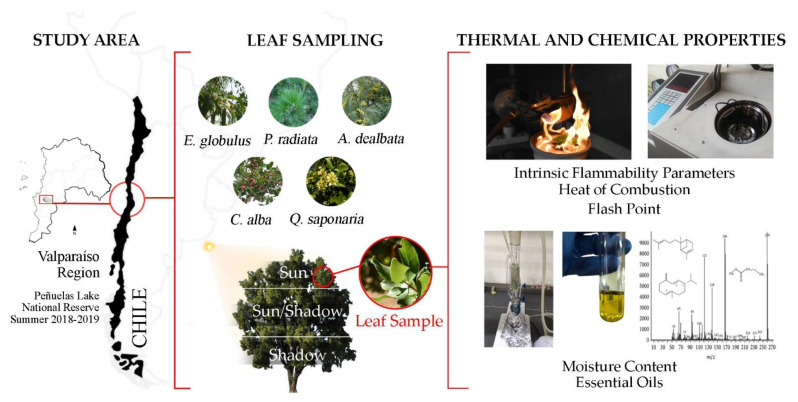
Schematic of the experimental procedure for the thermal and chemical characterization of exotic and native leaf samples.

For each species under study, 600 leaves were collected from three different sections of the tree according to sun exposure (200 leaves from each section of its canopy: sun, sun/shadow, and shadow (see Figure 3)), gathering a total of 3000 leaves per species, which were stored in hermetically sealed plastic bags to avoid damping. Each bag was labeled with the species name, section, and collection date. To maintain freshness, leaves were stored in a container with cooling gel before further analysis in the laboratory.

### 2.2. Flammability Measurements

To evaluate the flammability of the collected leaves, flammability tests were carried out using a methodology developed by various researchers [37,38,39,40,41,42,43,44,45,46,73,74], based on the use of a 500 W epiradiator (model 534 RC2, Quartz Alliance, France).

For each species, 50 leaf samples (each one of 1.0 ± 0.1 g) were placed on the surface of the epiradiator with a mean temperature of the radiative surface of 440 ± 9 °C with a coefficient of variation of 2%. Additionally, a pilot flame was added at a height of 4 cm above the radiative surface to ignite the mixture of volatile compounds resulting from the thermal degradation of the plant material. The parameters of ignition time (IT), flame duration (FD), and burning time (BT) were recorded, and an arithmetic mean of the 50 tests was then obtained for each tree species. Each procedure was carried out without direct handling, thus avoiding any modification of the leaf properties. The flammation frequency (Fr) for each species was calculated as the fraction of positive tests with respect to the total number of tests (*n* = 50). Tests were considered positive when flammation occurred in less than 1 min. If successive ignitions occurred and the duration of the first flame was equal to or less than 10 s, only the time of occurrence of the second ignition was considered for the validation of the test [37].

Across all tests, IT was considered as the time from the moment of contact between the leaf sample and the radiant surface of the epiradiator until the flammation of the plant material occurred. Subsequently, FD was taken as the time from flame ignition until its total disappearance. Finally, BT reflected the time required for each sample particle to be consumed, until the disappearance of the small embers. From these parameters, Valette’s flammability index (FI) [37] was used to classify the flammability of the leaves of each species. This index was calculated from the average IT and Fr values, (Table 1), and was a dimensionless parameter whose value ranged from zero (very low flammable) to five (extremely flammable) depending on the flammability of the material.

### 2.3. Heat of Combustion

The heating value is the amount of heat that is released in the complete combustion process per unit mass of fuel in an oxygen bomb calorimeter [54]. The higher heating value (HHV) and lower heating value (LHV) were obtained at a constant pressure by quantifying the change in the enthalpy of combustion with and without condensed water, respectively. The ASTM D240 procedure (a standard test method for the determination of the heat of combustion of liquid hydrocarbon fuels by a bomb calorimeter) [75] was used to determine both heating values for all leaf samples. Prior to determining the heating value, leaves were dried in a thermostatic oven at 110 ± 5 °C for 24 h and stored in a desiccator under environmental conditions until reaching room temperature. To obtain the HHV, dry samples (0.5 g) were burned in a Parr 1261 bomb calorimeter (Parr Instrument Co., Moline, IL, USA) operating under an isoperibolic process [75]. The HHV was calculated using Equation (1) as follows:

(1) $$\mathrm{HHV}=\frac{k\;\cdot \;\Delta T}{m}-Q$$

where HHV corresponds to the high heating value (MJ ${\mathrm{kg}}^{-1}$), k is the calorific capacity of the calorimeter (MJ$\;{}^{\circ}C^{-1}$), ΔT is the temperature difference obtained when heating the water cooling temperature (°C), m is the mass of dry leaves (kg), and Q is the heat released (MJ ${\mathrm{kg}}^{-1}$).

To obtain the LHV, dry samples (1.0 g) were burned in a Junkers calorimeter under adiabatic conditions [75]. In contrast to HHV, the steam generated from combustion was entrained in a trap with calcium chloride to absorb moisture and be quantifiable by mass difference. To calculate the heat of vaporization, the following equation was used:

(2) $$C_{v}=\frac{m_{c}\;\cdot \;h_{fg}}{m}$$

where $C_{v}$ corresponds to the heat of vaporization (MJ ${\mathrm{kg}}^{-1}$), $m_{c}$ is the mass of water adsorbed by the sodium chloride trap, $\;h_{fg}$ is the enthalpy of vaporization (MJ ${\mathrm{kg}}^{-1}$), and m corresponds to the mass of the burnt sample (kg). From the above, it was possible to obtain the LHV (MJ ${\mathrm{kg}}^{-1}$) using the following equation:

(3) $$\mathrm{LHV}=\mathrm{HHV}-C_{v}$$

### 2.4. Flash Point

The flash point (FP) is defined as the lowest temperature at which the vaporization of a volatile substance occurs, forming a flammable mixture with air in the presence of a continuous ignition source. According to ASTM D-92-72 (the standard test method for the determination of flash and fire points by a Cleveland open-cup), an open-cup Cleveland device was used, consisting of a heating dish, a thermometer (−6 to 400 °C), and an ignition source [76]. Leaves were added to the open-cup until it was filled and placed over the heating dish. The thermometer was vertically set over the cup with the tip positioned 6.6 mm from the bottom. Measurements were made at a heating rate of 15 °C min^−1^. The flash point was recorded as the temperature when a flame inside the cup caused a flash [6].

### 2.5. Chemical Extraction and Analysis

The leaves of the selected species were subjected to three extraction stages: Soxhlet extraction, liquid–liquid extraction, and concentration by rotavapor. Subsequently, the extracts were subjected to analysis by gas chromatography coupled to mass spectrometry (GC/MS). A mass sample of 17 g of leaves was weighed for each species and subjected to Soxhlet extraction with 80 mL of cyclohexane at 80 °C for 4 h. Subsequently, the extract solution was cooled to room temperature. To remove residual solvent, the extract solution was concentrated to dryness using a rotatory evaporator. This procedure was adapted from Ormeño et al., 2011 [77] and Wu et al., 2015 [78]. The concentrate from the Soxhlet extraction was transferred to a separation funnel where 80 mL of methanol and 50 mL of cyclohexane were added (this step was repeated twice, modifying the volumes). Two fractions were obtained for each species: one with polar compounds and the other with nonpolar compounds. The fractions were concentrated at room temperature to dryness and the final mass of each extract fraction was determined. The experimental error of the extraction process was measured as 5%.

The separation of the volatile (nonpolar compounds) and nonvolatile (polar compounds) phases was optimized based on the studies of Gonçalves et al., 2014 [79] and Jiang, 2005 [80]. Essential oil extraction (*EO*) yields were obtained by summing the polar and nonpolar phases. *EO* yield is expressed as the percentage of the essential oil weight according to Equation (4):

(4) $$EOs\left(\%w/w\right)=\frac{m_{EOs}}{m_{LV}}\cdot 100$$

where $m_{EOs}$ is the weight of essential oils obtained after extraction (g) and $m_{LV}$ is the weight of fresh leaf sample prior to extraction (g).

As extracts were obtained, the compounds present in the nonpolar fraction were analyzed and identified by GC/MS using a GC/MS-QP2010 Ultra combination (Shimadzu, Kyoto, Japan) equipped with an RTX-5MS nonpolar fused silica capillary column (30 m × 0.32 mm ID, 0.25 µm thickness; Restek, Bellafonte, PA, USA). The carrier gas was helium at a flow rate of 1 mL min^−1^. The oven was programmed from 50 °C (5 min hold) to 300 °C at 10 °C min^−1^ (30 min hold). The injection was carried out in split mode, and the injector temperature was 250 °C. The mass spectrometer operated in full-scan mode (scan range m/z 35–500, scanning frequency 0.3 s/scan) at 70 eV.

Compounds were identified by comparing their mass spectra and retention indices [81] with those reported in databases (NIST11 for MS, Waterman, 1996 [82] for retention indices).

### 2.6. Moisture Content

A mass of 10 g of fresh leaves was weighed on an analytical balance (AS 220.R2, Radwag) and placed in a thermostatic oven (digital drying oven, model JK-DO-9030A, JKI, China) at 110 °C for 24 h to ensure total loss of water. Afterward, samples were weighed and the moisture content (MC), defined as the weight of water as a function of dry weight, was determined according to Equation (5) [6,38,43,45,83,84]:

(5) $$MC\left(\%\right)=\frac{m_{LV}-m_{DR}}{m_{DR}}\cdot 100$$

where MC is the moisture content (%), $m_{LV}$ is the mass of the untreated leaf sample (g), and $m_{DR}$ is the mass of the dry leaf sample after the drying process (g).

In Figure 3, a schematic of the experimental apparatus used for the thermal and chemical characterization of exotic and native leaf samples collected in the PLNR is shown.

### 2.7. Data Analysis

Results from our investigations were expressed as the mean ± standard deviation together with the coefficient of variation. To analyze the normal distribution of flammability tests with 50 data points, the Kolmogorov–Smirnov normality test was used, and in cases where variables were not normal (6% of variables), these were transformed to comply with the assumptions of normality and homoscedasticity. In this way, the robustness of the parametric analyses was guaranteed. EO yields were presented as a mass concentration (%*w/w*), whereas the identified chemical compounds were expressed as percentages of relative area (RA%), indicating the percentage area for each identified compound, taking as a reference the sum of peak areas in each chromatogram. A simple linear regression analysis was performed to determine if there was a relationship between intrinsic flammability parameters (IT, FD, and BT; dependent variables: y), thermochemical properties (HHV, LHV, and FP; dependent/independent variables: y/x), and natural factors (MC and EOs) of leaf samples. The analysis aimed to establish if the flammability and thermochemical parameters could be estimated by natural factors in tree leaves. The analysis of variance was used with the following parameters: P: *p*-value, R: coefficient of correlation, and R^2^: coefficient of determination, with a confidence level of 95%.

An analysis of variance (ANOVA) was used to detect significant statistical differences between the means of each group and the HSD Tukey test was used to relate the statistical differences. For LHV and HHV, distribution tests and multiple comparison tests were also performed, including Fisher’s least significant difference (LSD), which uses mean values to prove the existence of statistically significant differences between groups. All statistical tests were performed using STATGRAPHIC Centurion XV software.

## 3. Results

### 3.1. Ignition Time

**Figure 4:**
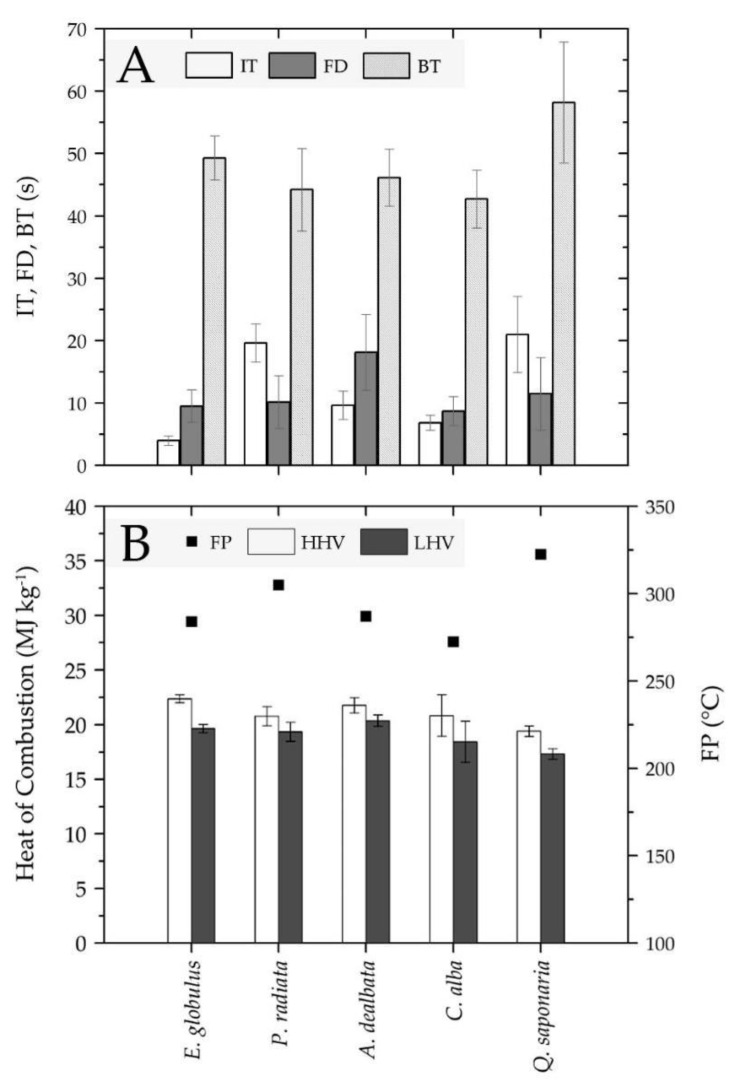
Intrinsic flammability parameters and thermochemical properties of leaf samples analyzed during summer 2018–2019: (**A**) ignition time (IT), flame duration (FD), and burning time (BT); (**B**) heat of combustion (HHV, LHV) and flash point (FP).

Results from the Kolmogorov–Smirnov test showed IT to be normally distributed with a 95% confidence interval for all analyzed forest species. Specifically, the following *p*-values were obtained: *Q. saponaria* (0.19), *C. alba* (0.10), *P. radiata* (0.20), *E. globulus* (0.20), and *A. dealbata* (0.19). The lowest IT was obtained for *E. globulus*, followed by *C. alba, A. dealbata, P. radiata*, and *Q. saponaria* (Figure 4A), with coefficients of variation of 18.58%, 16.99%, 23.40%, 15.51%, and 16.98%, respectively. Using ANOVA, the differences between the IT means were shown to be statistically significant due to the obtained *p*-value being less than 0.05 (*p* = 0.00), with a confidence level of 95%. In addition, the HSD Tukey test was used to compare significant differences between the means of the formed groups with a confidence level of 95% (see Table S1, Supplementary Materials). These results indicate the formation of nine significantly different pairs, while the members of the pair *P. radiata–Q. saponaria* were not significantly different from each other (*p* = 0.18).

### 3.2. Flame Duration

FD values were found to be normally distributed with a 95% confidence interval for all analyzed forest species, with *p*-values of 0.09, 0.20, 0.06, 0.06, and 0.20 obtained for *Q. saponaria*, *C. alba*, *P. radiata*, *E. globulus*, and *A. dealbata*, respectively. The highest FD was obtained for *A. dealbata*, followed by *Q. saponaria*, *P. radiata*, *E. globulus*, and *C. alba* (Figure 4A), with coefficients of variation of 33.52%, 50.56%, 42.53%, 27.32%, and 26.53%, respectively. Results from the ANOVA tests showed the differences between the means of the FD variable to be statistically significant between all five forest species, with a confidence level of 95%. Additionally, the HSD Tukey test was used to compare significant differences between the means of the formed groups with a confidence level of 95% (see Table S1, Supplementary Materials). The results indicated that the following pairs were significantly different: *Q. saponaria–C.alba* (*p* = 0.02); *Q. saponaria–A. delabata*; *C. alba–A. dealbata*; *E. globulus–A. albata*, and *P. radiata–A. dealbata* (all significances were *p* = 0.00).

### 3.3. Burning Time

BT values were found to be normally distributed with a 95% confidence interval for all analyzed forest species, and the following *p*-values were found: 0.20, 0.20, 0.20, 0.09, and 0.20, for *Q. saponaria*, *C. alba*, *P. radiata*, *E. globulus*, and *A. dealbata*, respectively. The highest BT was obtained for *Q. saponaria*, followed by *E. globulus*, *A. dealbata*, *P. radiata*, and *C. alba* (Figure 4A). The coefficients of variation obtained for forest species *Q. saponaria, C. alba, P. radiata, E. globulus, and A. dealbata* were 16.67%, 10.62%, 14.95%, 7.18%, and 9.90%, respectively. Using ANOVA, differences between the means of the BT variable were shown to be statistically significant due to the obtained *p*-value being less than 0.05 (*p* = 0.00), with a confidence level of 95%. In addition, the HSD Tukey test showed that the following pairs presented significant differences with each other: *Q. saponaria–C. alba*, *Q. saponaria–E. globulus*, *Q. saponaria–P.radiata*, *Q. saponaria–A. dealbata*, *C. alba–E. globulus* (all significances were *p* = 0.00), and *E. globulus–P. radiata* (*p* = 0.00) (see Table S1, Supplementary Materials).

### 3.4. Flammability Index

The leaves of the studied forest species were classified based on the flammability index proposed by Valette [37]. All sample species obtained a Fr of 100% across all 50 tests. Forest species *C. alba, E. globulus*, and *A. dealbata* obtained an FI of 5 and therefore were classified as extremely flammable, while *Q. saponaria* and *P. radiata* obtained an FI of 3, classifying them as flammable species (Table 2).

### 3.5. Heat of Combustion

The obtained results were adjusted to a normal distribution with a 95% confidence interval, showing significant differences between the means of each HHV and LHV with *p*-values of less than 0.05 (*p* = 0.00). The highest HHV was obtained by *E. globulus*, followed by *A. dealbata, C. alba, P. radiata*, and *Q. saponaria* (Figure 4B). Each HHV test was performed in duplicate, and the coefficients of variation obtained for *E. globulus, A. dealbata, C. alba, P. radiata*, and *Q. saponaria* were 0.24%, 0.96%, 0.37%, 0.47%, and 0.21%, respectively. Multiple range tests for HHV showed that only the *C. alba–P. radiata* pair did not exhibit a significant difference (difference = 0.06 MJ ${\mathrm{kg}}^{-1}$). By comparison, the *E. globulus–Q. saponaria* pair had the maximum significant difference of 2.98 MJ ${\mathrm{kg}}^{-1}$ (Table S2).

In the case of LHVs, the multiple range tests showed statistically significant differences between all LHV values, with the pair *A. dealbata–Q. saponaria* having the highest significant difference of 3.06 MJ ${\mathrm{kg}}^{-1}$. All tests were performed with a 95% confidence interval. The highest LHV was obtained for the forest species *A. dealbata*, followed by *E. globulus, P. radiata, C. alba*, and *Q. saponaria* (Figure 4B), resulting in coefficients of variation of 1.03%, 0.27%, 0.51%, 0.31%, and 0.24%, respectively (*n* = 2) (see Table S2, Supplementary Materials).

### 3.6. Flash Point

The obtained results were adjusted to a normal distribution with a confidence interval of 95%, showing no significant differences between the means of each FP and a *p*-value greater than 0.05 (*p* = 0.16). The lowest FP registered was found for species *C. alba*, followed by *E. globulus*, *A. dealbata*, *P. radiata*, and *Q. saponaria* (Figure 4B), while the coefficients of variation were 1.29%, 6.97%, 1.22%, 6.95%, and 7.67%, respectively. HSD Tukey tests did not show significant differences between pairs of species with a confidence level of 95% (see Table S2, Supplementary Materials).

### 3.7. Moisture Content

**Figure 5:**
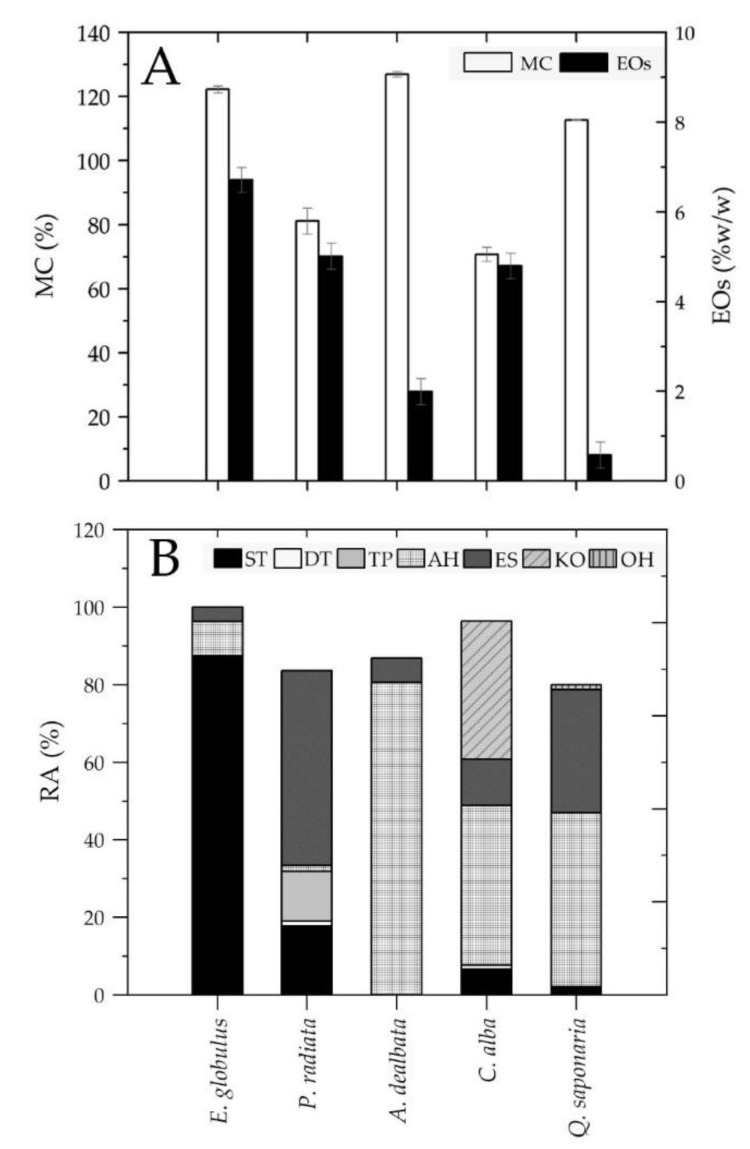
Natural factors analyzed during summer 2018–2019: (**A**) moisture content and essential oil content, and (**B**) main chemical compounds found in EO extracts expressed as relative area (RA%).

The obtained results were adjusted to a normal distribution with a confidence interval of 95%, showing no significant differences between the means of each MC and a *p*-value less than 0.05 (*p* = 0.000). Leaf samples of *A. dealbata* recorded the highest MC value among all species under study, followed by *E. globulus*, *Q. saponaria*, *P. radiata*, and *C. alba* (Figure 5A). In addition, the HSD Tukey test showed that the following groups presented significant differences with each other between the means of each MC with a confidence level of 95%: *A. delabata–C. alba*, *A. dealbata–P. radiata*, *A. dealbata–Q. saponaria*, *E. globulus–C. alba*, *E. globulus–P. radiata*, *C. alba–P. radiata*, *C. alba–Q. saponaria*, and *P. radiata–Q. saponaria*. By contrast, the groups *A. dealbata–E. globulus* and *E. globulus–Q. saponaria* did not present significant differences between the means of each MC, with a confidence level of 95% (see Table S2, Supplementary Materials).

### 3.8. Essential Oils and Identification of Terpenes/Other Chemical Compounds in Leaves

Figure 5A shows the content of EOs in the leaves of the forest species under study, where *E. globulus* had the highest concentration of EOs, followed by *P. radiata*, *C. alba*, *A. dealbata*, and *Q. saponaria*. A total of 58 chemical compounds were identified comprising sesquiterpenes (ST), aliphatic hydrocarbons (AH), esters (ES), ketones (KO), alcohols (OH), diterpenes (DT), and triterpenes (TP), as shown in Figure 5B. Differences in predominant compound classes were observed according to each species. The highest terpene content was found in *E. globulus* with 87% of ST, while AH was dominant in EOs of *A. dealbata* and *Q. saponaria*, reaching levels of 81% and 45%, respectively. In contrast, ES represented 50% of the EO concentration in *P. radiata*, while KO was mainly present in *C. alba* samples, reaching 36% of the relative area (RA). The major compounds identified were alloaromadendrene (ST; 47.37%), methyl dodecanoate (ES; 27.8%), nonacosane (AH; 78.4%), 16-hentriacontanone (KO; 35.6%), and nonacosane (AH; 31.99%) for *E. globulus*, *P.radiata*, *A. dealbata*, *C. alba*, and *Q. saponaria*, respectively (see Table S3, Supplementary Materials).

### 3.9. Relationship between Thermochemical Parameters and Natural Drivers of the Leaves

**Figure 6:**
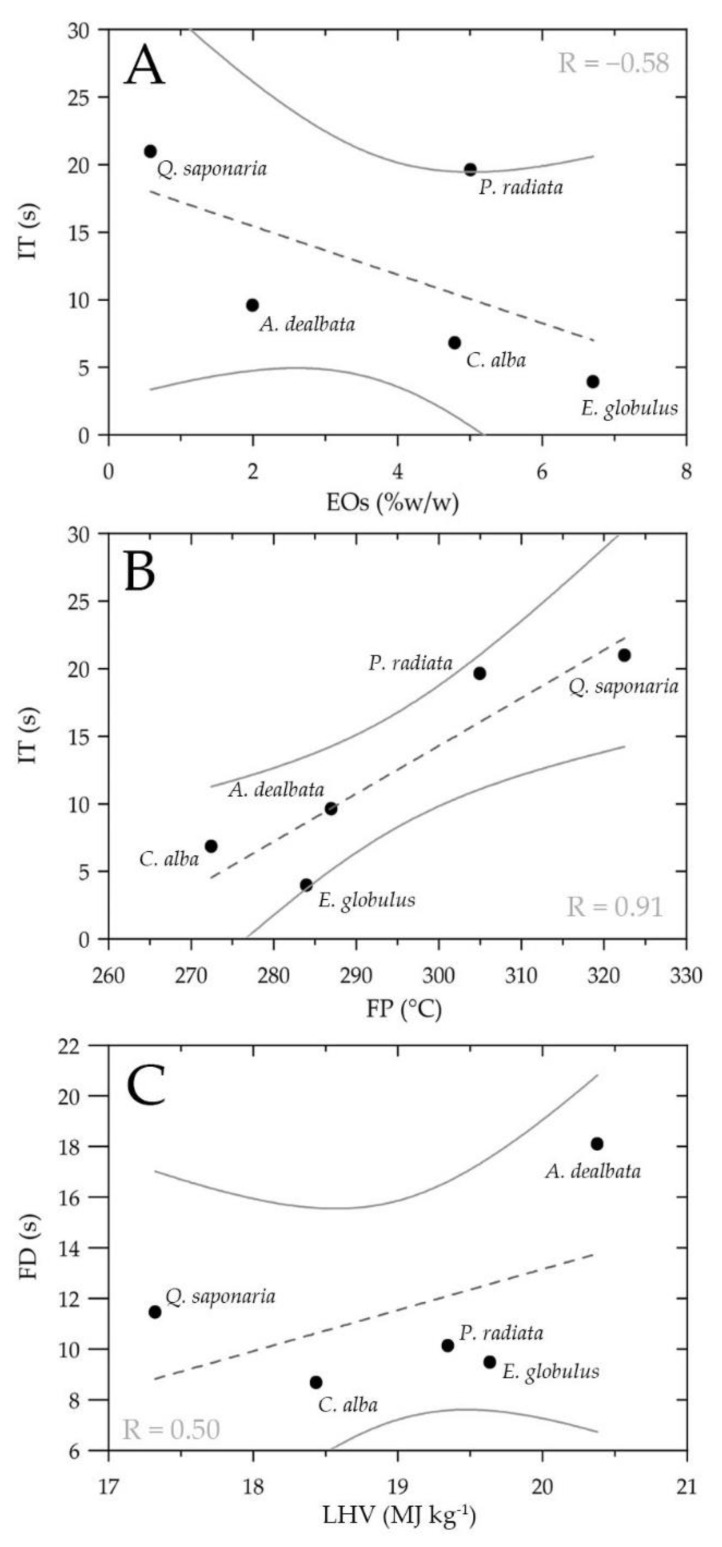
Relationship between essential oil (EO) concentration, thermochemical properties (FP and LHV), and intrinsic flammability parameters of the ignition time (IT) and flame duration (FD) of leaf samples. Simple regression analysis between: (**A**) EOs and IT, (**B**) IT and FP, and (**C**) FD and LHV.

Figure 6 shows the main relationships obtained between the EO concentration, thermochemical properties (FP and LHV), and intrinsic flammability parameters of the IT and FD of leaf samples. The EOs showed a moderately strong negative correlation with the IT parameter (R = −0.58) (Figure 6A), i.e., as the concentration of EOs contained in the leaves increases, there is a decrease in the ignition time, leading to an increase in flammability. Furthermore, the FP showed a relatively strong positive correlation with the IT parameter (R = −0.91) (Figure 6B); as the flash point of leaves increases, there is an increase in the ignition time (decrease in flammability). On the other hand, the LHV showed a moderately strong positive correlation (R = −0.50) with the FD parameter (Figure 6C), implying that as the energy released by the leaves in a combustion process increases, an increase in flame duration is generated.

Other relationships can be found in Table S4 (Supplementary Materials), from which we highlight that moisture content showed a weak negative correlation (R = −0.15) with the IT parameter, which is contradictory to results found in other research [45,84,85], where MC significantly affected leaf IT (positive effect), confirming that leaves with a high moisture content took longer to ignite.

## 4. Discussion

### 4.1. Variation in Flammability of Forest Species

Based on the arithmetic mean of IT, our results suggest that tree species *E. globulus* and *C. alba* showed a higher predisposition to foliage ignition and were therefore more vulnerable to the initiation of crown fire events. In contrast, the least vulnerable species to foliage ignition were *Q. saponaria* and *P. radiata*.

It is possible to characterize the time at which the combustion energy is irradiated with greater intensity after ignition through the FD parameter; therefore, in a foliage fire, we would expect that the species *A. dealbata* and *Q. saponaria* would be at the most risk of propagation by irradiation when compared to the arithmetic mean of the FD of this irradiation. Specimens of *C. alba* and *E. globulus* showed the lowest mean FD of this parameter. Similarly, the parameter BT can represent the time at which incandescent material exists and could represent a source of energy for new ignitions. In this sense, the species *Q. Saponaria* and *E. globulus* had the highest arithmetic means of BT, in contrast to *C. alba* and *P. radiata*, which presented the lowest arithmetic means of BT.

The major contribution of our research is represented by the flammability parameters obtained for the native species *C. alba* and *Q. saponaria*, as no previous study in Chile has reported these parameters using Valette’s classification [37]. A remarkable result is related to the species *C. alba*, which obtained a low IT of 6.85 ± 1.16 s (FI = 5: extremely flammable), similar to the exotic forest species *E. globulus* with an IT parameter of 3.98 ± 0.74 s. If these species are in front of an ignition source that does not require high heat fluxes (cigarette butts, sparks, poorly extinguished campfires, etc.), the species will ignite faster, starting crown fires.

No specimens were found to be non-flammable according to Valette’s classification, which together with the values obtained for IT, FD, and BT parameters allow us to estimate the limitations that some individuals of native species could present for consideration in the creation of landscapes that are resilient to climate change and its effects [86]. The values of the thermophysical properties show that both native and exotic species can be risky and vulnerable to heat sources, fire spread, and prolonged fire persistence during fire events.

It must be pointed out that the obtained results have limitations in terms of being scaled to landscape levels, since the heat transfer processes in experiments are different from those occurring in an uncontrolled environment [43,44], and we did not consider climatic variables such as those by the National Forestry Corporation CONAF (ignition probability index as a wildfire prevention strategy). However, characterizing fuels with standardized scientific parameters allows us to estimate the behavior of vegetation in wildfires, as well as to identify the vulnerability or danger of plant species on a quantitative basis. In this regard, our results were consistent with those reported by other studies carried out in different places with similar climatic conditions with exotic species (*E. globulus, P. radiata*, and *A. dealbata*) [39,44,87,88].

### 4.2. Variation of Heating Values

Statistically significant differences were found regarding HHV and LHV for all forest species investigated in our study. This can be attributed to the fact that the heat generated from forest fuel is related to the chemical composition, structure, and moisture content of the fuel [51].

The results from our study regarding HHV correspond to those reported by Guerrero et al., 2020 [6] and, in order to follow the same classification criteria used in their study, our study species were classified as follows: exotic species *E. globulus* and *A. dealbata* were categorized as Class 5, *P. radiate* as Class 4, native species *C. alba* as Class 4, and *Q. saponaria* as Class 2 based on their heat levels at combustion. Notably, the exotic species had the highest HHV, with *E. globulus* and *P. radiata* previously reported to have a high HHV [89]. However, the native species *C. alba* obtained a similar value to *P. radiata*, indicating it to be a high-risk species.

Regarding LHV, our results showed higher values for the majority of analyzed species compared to those reported by Guerrero et al. [6], with the exception of *C. alba*, where a slight decrease was obtained compared to the 2017–2018 summer. This increase in LHV possibly indicates the presence of an external influencing factor, although the heating value varies according to species and according to, among others, the energy value of EOs, lignocellulosic material, and the MC of the fuel [51]. Therefore, water stress, periods of drought, and heat waves registered in Valparaíso and the central–southern regions of Chile could affect the thermochemical properties of forest fuel. In this context, various studies have highlighted the ability of certain species to increase the content of chemical compounds in response to abiotic factors as a protection mechanism and physiological adaptations, for example, the increase in essential oils [85,90].

The forest species *A. dealbata* and *E. globulus* had the highest LHV value of 20.38 ± 0.21 MJ ${\mathrm{kg}}^{-1}$ and 19.64 ± 0.05 MJ ${\mathrm{kg}}^{-1}$, respectively, compared to *Q. saponaria* with the lowest LHV of 17.32 ± 0.04 MJ ${\mathrm{kg}}^{-1}$. As LHV is obtained during combustion in open air when water evaporates to the environment (Byram intensity) [54], it represents usable heat, and therefore *A. dealbata* and *E. globulus* are more likely to generate high-intensity fires. In comparison, *Q. saponaria*, which had the lowest LHV, represents a lower risk as a heat source than species with a higher LHV. Additionally, when comparing FD to LHV (Figure 6C), a moderately strong positive relationship between these parameters is observed, i.e., as LHV increases, an increasing trend in FD is observed. In particular, the species *A. dealbata* may generate a higher risk of fire spread due to the fact that, in the flame phase, it releases a high amount of energy for a longer period of time compared to other tree species.

Finally, our study focuses on flammability and heating value, two thermochemical properties that should not be viewed as independent components, but rather as a group of characteristics related to certain forest species that can be investigated in association with other variables such as climatic conditions, diversity, density, and geography. For example, the forest species *E. globulus* and *A. dealbata* were classified as extremely flammable (FI = 5) and obtained the highest LHV and HHV (extremely high risk), while the native species *C. alba* was also found to be an extremely flammable species (FI = 5) and was classified as a high-risk species according to its HHV. Based on these results, the three species have a high predisposition to ignite in the presence of a heat source. Furthermore, they have a high risk of becoming heat sources, which contributes to the overall effect of forest fires. In contrast, *Q. saponaria* showed interesting results as it was the least flammable species (flammable, FI = 3) and had the lowest LHV and HHV (low risk); therefore, it is a species that could reduce the occurrence, spread, and effects of forest fires.

### 4.3. Drivers of Leaf Flammability: Essential Oils, Chemical Compounds, and Moisture Content

The results obtained show that the concentration of EOs was the most relevant natural factor due to the negative and moderately strong relationship with the IT parameter, i.e., increased concentrations of EOs in the leaves can result in a decrease in IT and thus generate an increase in the flammability of the vegetation. For example, *E. globulus* had the lowest IT and highest FI compared to the other exotic species. This could be explained based on our results, which showed that this species had the highest content of EOs (6.71 ± 0.28% *w/w*), composed mainly of sesquiterpenes (87.35% RA), highlighting a high presence of alloaromadendrene, which has an FP of 106.5 °C. This indicates that, in the presence of an ignition source, the species will ignite at a low temperature and concentration [85]. Other studies [45,74] have reported that leaves of the genus eucalyptus (*E. globulus* and *E. camaldulensis*) are highly flammable due to the high content of EOs in their leaves; thus, it acts as a fire enhancer, accelerating the ignition time [91]. The oils are found in, among others, the bark, branches, leaves, and flowers of the plant, and consist of chemical compounds with low flash points, such as terpenes (monoterpenes and sesquiterpenes), for example, eucalyptol, a monoterpene that is the main component extracted from eucalyptus leaves and has a low FP (FP = 49 °C) [92]. In addition, high rates of isoprene and monoterpene emissions have been identified in eucalyptus trees [93], generating a flammable atmosphere that can contribute to the formation of forest fires.

Regarding the native tree species, the high flammability of *C. alba* can be attributed to the fact that it registered a high concentration of EOs (4.90 ± 0.28%). Moreover, this species presented several highly flammable sesquiterpenes, where a very particular chemical compound called 16-hentriacontanone was identified, a ketone with a very low FP (35.6 °C). We think this compound could give flammable properties to *C. alba* leaves. In addition, flammable chemical compounds (monoterpenes and sesquiterpenes) have been reported for *C. alba*, for example α-terpineol, eucalyptol, phellandrene, terpinen-4-ol, and p-cymene with flash points of 88, 51, 47, 79, and 47 °C, respectively [94,95]. In contrast, the native species *Q. saponaria* showed the highest IT. This could be attributed to its low EO content (0.58 ± 0.29% *w/w*), which was 12- and 8-times lower than *E. globulus* and *C. alba*, respectively. Furthermore, aliphatic hydrocarbons of higher FP were mainly identified in the EOs of *Q. saponaria*, representing a possible factor capable of explaining its low flammability. These differences in flammability between native species may indicate that *Q. saponaria* can develop a more adapted structure in response to natural conditions due to drought and high temperatures, such as an increase in the production of aliphatic hydrocarbons (higher FP) or an increase in the thickness of the epidermis (characteristics of sclerophyllous leaves), which possibly allow greater water retention and chemical compounds, making the plant less flammable.

It is important to highlight the forest species *A. dealbata*, which had the second lowest IT and can be classified as extremely flammable (FI = 5), despite having a three times lower content of EOs (2.26 ± 0.29% *w/w*) compared to *E. globulus*, where monoterpenes and sesquiterpenes with low flash points were not identified, and it was the first species with the highest MC (127 ± 1.41%) among all species under study. This could be related to the fact that other plant characteristics can affect flammability; for example, Murray et al., 2013 [96] showed that a low IT (high flammability) in species of the genus Acacia (*A. linifolia*, *A. longifolia*, *A. suaveolens*, *A. ulicifolia*, and *A. terminalis*) had a strong relationship with the large size of leaves. However, this study shows that it is important to consider other parameters such as the thickness of the cuticles contained in the upper layer of the leaves. Another factor to consider is the phenological state of the leaf, as was studied by Valette, 1990 [37], who reported similar results to ours, but in phases where *A. dealbata* leaves are growing and hardened (FI = 4–5), while the leaves in their mature phase registered a lower flammability (FI = 1–4). Another report by Hachmi et al., 2011 [39] showed that the same genus and species (Acacia mollissima) is extremely flammable, despite having a MC higher than 140%, which is attributed to the existence of other natural factors, such as EOs that can interact with the MC.

The species *P. radiata* was the least flammable of the exotic species (FI = 3); however, it registered a low MC (81 ± 4.24%), a high content of EOs (5.01 ± 0.29% *w/w*), and a diversity of sesquiterpenes characterized by low flash points such as (E)-β-caryophyllene, α-bergamotene, cis-α-bisabolene, alloaromadendrene, germacrene D, α-farnesene, and α-chamigrene. This confirms, as was in the case of *A. dealbata*, that other interspecific factors of leaves play a fundamental role in flammability, principally the leaf anatomy and, in the case of *P. radiata*, the leaf shape (elongated and rigid needles). This can be explained as described by De Lillis et al. 2009 [97], where it was demonstrated that the needles of *Pinus halapensis*, a highly resinous conifer tree species, are a weak emitter of monoterpene, but are capable of storing a large amount of isoprenoids, which are released only when temperatures are very high. This occurs because there are resistant structures that surround the pine resin ducts, leaving them hermetically sealed, and this possibly cancels the effect of terpenes on flammability.

In summary, EOs are a complex mixture of chemical compounds comprising mostly low-molecular-mass volatile organic compounds (under 300 g mol^−1^), such as hydrocarbons, alcohols, ethers, aldehydes, ketones, esters, amines, amides, and phenols. Terpenes (monoterpenes and sesquiterpenes), in particular, have low flash points [98]. Therefore, these EOs can act as fire drivers that increase the flammability of forest species’ leaves. Dimitrakopoulos and Papaioannou, 2001 [91] showed that the leaves of the Mediterranean forest species *Laurus nobilis* and *Eucalyptus camaldulensis* were extremely flammable due to their high contents of flammable volatile compounds present in EOs. Thus, forest fuels rich in volatile essential oils (e.g., Eucalyptus, Laurel, Pinus, and Pistacia) are flammable due to their low IT [91].

In particular, our MC results were similar to those reported by Bianchi et al., 2019 [84], Ganteaume, 2018 [45] and Grootemaat et al., 2015 [99], with results of between 72–253%, 72–213%, and 68–231% respectively. These results can be attributed to differences in the physiological processes, photosynthetic capacity, and carbon storage of leaves, which change the biochemical components of dry matter, such as crude fat (essential oils and impurities) and structural or nonstructural compounds that cause temporary changes in the MC [100,101].

In addition to the natural factors aforementioned, it is important to highlight the positive relationship between the FP and IT parameters, where it can be noticed that as FP is reduced, there is a decrease in the IT. This relationship can be attributed to the identification of low FP chemical compounds found in EOs. Low flash point chemical compounds (mainly monoterpenes and ketones) were identified in the different exotic and native species: aromadendrene (ST, FP: 106 °C) in *E. globulus*; (E)-β-caryophyllene (ST, FP: 104 °C) in *P. radiata*; nonacosane (AH, FP: 291 °C) in *A. dealbata*; heptacosane (AH, FP: 268 °C) in *C. alba*; and hexadecane (AH, FP: 135 °C) in *Q. saponaria*. Such terpenoids and aliphatic hydrocarbons are highly flammable; therefore, when accumulated in leaves and in the presence of an ignition source, they would ignite at relatively low temperatures and concentrations [57].

## 5. Conclusions

This research has provided new knowledge on the classification of forest species constantly affected by forest fires based on the flammability index proposed by Valette [37]. Regarding the evaluated tree species, all are flammable (*Q. saponaria*, *P. radiata*) and in some cases extremely flammable (*C. alba*, *E. globulus*, *A. delabata*); however, differences are observed both in the predisposition to ignition (IT ratio, EOs, FP) and in the ability to spread fire by irradiation (FD and LHV ratio). Furthermore, it was shown that the high concentration of EOs contained in the leaves decreases the ignition time, and, therefore, they can be considered as flammability drivers in the predominant species of the Mediterranean climate.

From the analysis of the relationships between the intrinsic properties of flammability, thermochemistry, and the concentration and composition of essential oils, it is not possible to affirm that there are differences in the flammability of native species when compared to exotic species without distinction. In contrast, there are relevant differences between the behaviors of the species *C. alba* (extremely flammable) and *Q. saponaria* (flammable).

It must be emphasized that the results presented in this study contribute to the establishment of a flammability classification according to the evolution of the risk of fire initiation presented by the species, taking into account their role in the different forest formations in the country. In this regard, this information will help to (i) integrate flammability criteria into fire risk indices, (ii) establish risk charts based on vegetation maps, and (iii) implement green barriers as a tool for reducing the risk of damage to vulnerable areas such as urban–forest interfaces and conservation areas.

Based on our results, we recommend that organizations dedicated to the management and handling of forest fires, primarily the National Forestry Corporation (CONAF), focus on the development of preventive measures that consider the high flammability and energy content of the forest species *C. alba*, *E. globulus*, and *A. dealbata*.

Finally, future research should consider an extension of the geographical and temporal scale of study, expanding the sampling area to other regions of south-central Chile frequently affected by forest fires, incorporating thermal and chemical characterization of leaf litter, and systematically carrying out analyses over time in order to develop a more representative flammability classification based on natural factors of Mediterranean forest vegetation.

## Figures and Tables

**Table 1:** Flammability index ^1^ (FI) as a function of flammation frequency (Fr) and mean ignition time (IT).

IT (s)	Fr (%)				
	100–95	94–90	89–85	84–80	< 50
**< 12.5**	5	4	3	3	1
**12.5–17.5**	4	3	3	2	1
**17.5–22.5**	3	3	2	2	0
**22.5–27.5**	3	2	2	1	0
**27.5–32.5**	3	2	2	1	0
**>32.5**	3	1	2	0	0

^1^ Valette´s classification [37]: very slightly flammable (FI = 0), slightly flammable (FI = 1), moderately flammable (FI = 2), flammable (FI = 3), very flammable (FI = 4), and extremely flammable (FI = 5).

**Table 2:** Classification of the flammability of forest species according to flammability index (FI), based on the average ignition time (IT) and flammation frequency (Fr).

Species	IT (s) (x±s)	Fr (%)	FI (-)	Classification
***E. globulus***	3.98 ± 0.74	100	5	Extremely flammable
***C. alba***	6.85 ± 1.16	100	5	Extremely flammable
***A. dealbata***	9.65 ± 2.26	100	5	Extremely flammable
***P. radiata***	19.64 ± 3.08	100	3	Flammable
***Q. saponaria***	20.99 ± 6.09	100	3	Flammable

## Data Availability

The data presented in this study are available on request from the corresponding author.

## Supplementary Materials

The following are available online at https://www.mdpi.com/article/10.3390/ijerph18137191/s1, Table S1: Multiple comparisons using the HSD Tukey test of flammability parameters of ignition time (IT), flame duration (FD), and total burning time (BT), Table S2: Multiple range test through the percent LSD/HSD test with the confidence interval at 95% of higher heating value (HHV), lower heating value (LHV), flash point (FP), and moisture content (MC), Table S3: Identification of organic compounds by chromatography (GC/MS) contained in the essential oils (EOs) of exotic and native species in summer 2018–2019, Table S4: Simple regression analysis between parameters of flammability, heating values, and natural driver of leaves with a confidence level of 95%. EOs and MC, independent variables: x; IT, FD and BT, dependent variables: y; and heating value, HHV and LHV, dependent/independent variables: y/x.

## References

[1] Costafreda-Aumedes S., Comas C., Vega-Garcia C. (2017). Human-caused fire occurrence modelling in perspective: A review. Int. J. Wildl. Fire.

[2] Reisen F., Duran S.M., Flannigan M., Elliott C., Rideout K. (2015). Wildfire smoke and public health risk. Int. J. Wildl. Fire.

[3] Cascio W.E. (2018). Wildland fire smoke and human health. Sci. Total. Environ..

[4] Fann N., Alman B., Broome R.A., Morgan G.G., Johnston F.H., Pouliot G., Rappold A.G. (2018). The health impacts and economic value of wildland fire episodes in the U.S.: 2008–2012. Sci. Total Environ..

[5] Finlay S., Moffat A.J., Gazzard R., Baker D., Murray V. (2012). Wildfires and Health Impacts. PLoS Curr. Disasters.

[6] Guerrero F., Toledo M., Ripoll N., Espinoza L., Morales R., Muñoz A., Taborga L., Carrasco Y. (2020). Thermo-and physicochemical properties of native and exotic forest species of Valparaíso, Chile, as essential information for fire risk management. Int. J. Wildl. Fire.

[7] Hodzic A., Madronich S., Bonn B., Massie S., Menut L., Wiedinmyer C. (2007). Wildfire particulate matter in Europe during summer 2003: Meso-scale modeling of smoke emissions, transport and radiative effects. Atmos. Chem. Phys..

[8] Sitch S., Cox P.M., Collins W.J., Huntingford C. (2007). Indirect radiative forcing of climate change through ozone effects on the land-carbon sink. Nature.

[9] Miranda A.I. (2004). An integrated numerical system to estimate air quality effects of forest fires. Int. J. Wildl. Fire.

[10] Green B.L. (1991). Evaluating the Effects of Disasters. Psychol. Assess..

[11] Ojeda C.G., Jaque Castillo E., Fernández Castillo S. (2021). Postwildfire Landscape Identity in Mediterranean Ecosystems: Three Study Cases from the Coastal Range of Central Chile. Ann. Am. Assoc. Geogr..

[12] Carmona C.P., Azcárate F.M., de Bello F., Ollero H.S., Lepš J., Peco B. (2012). Taxonomical and functional diversity turnover in Mediterranean grasslands: Interactions between grazing, habitat type and rainfall. J. Appl. Ecol..

[13] Keeley J.E., Bond W.J., Bradstock R.A., Pausas J.G., Rundel P.W. (2011). Fire in Mediterranean Ecosystems.

[14] Turco M., Von Hardenberg J., AghaKouchak A., Llasat M.C., Provenzale A., Trigo R.M. (2017). On the key role of droughts in the dynamics of summer fires in Mediterranean Europe. Sci. Rep..

[15] Smith C.W., Panda S.K., Bhatt U.S., Meyer F.J. (2021). Improved boreal forest wildfire fuel type mapping in interior alaska using aviris-ng hyperspectral data. Remote Sens..

[16] CALFIRE California Department of Forestry and Fire Protection. https://calfire.ca.gov.

[17] Goss M., Swain D.L., Abatzoglou J.T., Sarhadi A., Kolden C.A., Williams A.P., Diffenbaugh N.S. (2020). Climate change is increasing the likelihood of extreme autumn wildfire conditions across California. Environ. Res. Lett..

[18] Yu P., Xu R., Abramson M.J., Li S., Guo Y. (2020). Bushfires in Australia: A serious health emergency under climate change. Lancet Planet Heal..

[19] Soto M.C., Julio-Alvear G., Salinas R.G., Shroder J.F., Paton D. (2015). Current Wildfire Risk Status and Forecast in Chile: Progress and Future Challenges. Wildfire Hazards, Risks, and Disasters.

[20] Vicencio J., Cortés C., Campos D.E., Tudela V. (2017). Informe Climático Especial Enero 2017: Un mes de récords. Sección Meteorología Agrícola. Sección Climatología.

[21] De la Barrera F., Barraza F., Favier P., Ruiz V., Quense J. (2018). Megafires in Chile 2017: Monitoring multiscale environmental impacts of burned ecosystems. Sci. Total Environ..

[22] (2011). FAO—Food and Agriculture Organization Wildfire Prevention in the Mediterranean. http://www.fao.org/3/i2495e/i2495e00.htm.

[23] CONAF Incendios Forestales en Chile, Estadísticas Históricas: Resumen Nacional y Regional de Ocurrencia (Número) y Daño (Superficie Afectada) por Incendios Forestales. 1964–2019. www.conaf.cl/incendios-forestales/incendios-forestales-en-chile/estadisticas-historicas/.

[24] Bowman D.M.J.S., Moreira-Muñoz A., Kolden C.A., Chávez R.O., Muñoz A.A., Salinas F., González-Reyes Á., Rocco R., de la Barrera F., Williamson G.J. (2019). Human–environmental drivers and impacts of the globally extreme 2017 Chilean fires. Ambio.

[25] CONAF Fondo de Investigación del Bosque Nativo Aporta a la Recuperación del Bosque Esclerófilo. www.conaf.cl/fondo-de-investigacion-del-bosque-nativo-aporta-a-la-recuperacion-del-bosque-esclerofilo/.

[26] CONAF Controlan Extenso Incendio Forestal Que Afectó a Valparaíso y Quilpué. www.conaf.cl/controlan-extenso-incendio-forestal-que-afecto-a-valparaiso-y-quilpue/.

[27] Bowman D.M.J.S., Williamson G.J., Abatzoglou J.T., Kolden C.A., Cochrane M.A., Smith A.M.S. (2017). Human exposure and sensitivity to globally extreme wildfire events. Nat. Ecol. Evol..

[28] Garreaud R.D., Boisier J.P., Rondanelli R., Montecinos A., Sepúlveda H.H., Veloso-Aguila D. (2020). The Central Chile Mega Drought (2010–2018): A climate dynamics perspective. Int. J. Climatol..

[29] Castellnou M., Álvarez R., Almodóvar J., Martins F., Mayoral P.C., Alfonso L., Basile G. (2017). Informe Técnico: Situación de Incendios Forestales en Chile entre Enero-Febrero 2017.

[30] Garreaud R.D., Alvarez-Garreton C., Barichivich J., Pablo Boisier J., Christie D., Galleguillos M., LeQuesne C., McPhee J., Zambrano-Bigiarini M. (2017). The 2010-2015 megadrought in central Chile: Impacts on regional hydroclimate and vegetation. Hydrol. Earth Syst. Sci..

[31] CEPAL (2012). La economía del cambio climático en Chile. Com. Economica America Lat. Caribe.

[32] Araya-Osses D., Casanueva A., Román-Figueroa C., Uribe J.M., Paneque M. (2020). Climate change projections of temperature and precipitation in Chile based on statistical downscaling. Clim. Dyn..

[33] Urrutia-Jalabert R., González M.E., González-Reyes Á., Lara A., Garreaud R. (2018). Climate variability and forest fires in central and south-central Chile. Ecosphere.

[34] Anderson H.E. (1970). Forest fuel ignitibility. Fire Technol..

[35] Martin R.E., Gordon D.A., Gutierrez M.A., Lee D.S., Domingo M.M.T., Schroeder R.A., Sapsis D.B., Stephens S.L. Assessing the Flammability of Domestic and Wildland Vegetation. Proceedings of the 12th Conference of Fire and Forest Meteorology.

[36] White R.H., Zipperer W.C. (2010). Testing and classification of individual plants for fire behaviour: Plant selection for the wildlandurban interface. Int. J. Wildl. Fire.

[37] Valette J.-C. (1990). Inflammabilités des espèces forestières méditerranéennes: Conséquences sur la combustibilité des formations forestières. Rev. For. Fr..

[38] Ormeño E., Céspedes B., Sánchez I.A., Velasco-García A., Moreno J.M., Fernandez C., Baldy V. (2009). The relationship between terpenes and flammability of leaf litter. For. Ecol. Manag..

[39] Hachmi M., Sesbou A., Benjelloun H., El Handouz N., Bouanane F. (2011). A simple technique to estimate the flammability index of moroccan forest fuels. J. Combust..

[40] Batista A., Biondi D., Tetto A., Assunção R. (2012). De Evaluación de la Inflamabilidad de Árboles y Arbustos Utilizados en la Implementación de Barreras Verdes en el Sur del Brasil. Proceedings of the Fourth International Symposium on Fire Economics, Planning, and Policy: Climate Change and Wildfires, Mexico, USA, 5–11 November 2021.

[41] Frejaville T., Curt T., Carcaillet C. (2013). Bark flammability as a fire-response trait for subalpine trees. Front. Plant Sci..

[42] Della Rocca G., Madrigal J., Marchi E., Michelozzi M., Moya B., Danti R. (2017). Relevance of terpenoids on flammability of Mediterranean species: An experimental approach at a low radiant heat flux. IForest Biogeosci. For..

[43] Rodríguez Y.C., Rodríguez M.P.R., Mesa F.J., Hernández Y.C., Becerra L.W.M. (2016). Inflamabilidad de especies vegetales del ecosistema de pinares. Rev. Cuba. Ciencias For..

[44] Molina J.R., Martín T., Rodríguez Y., Silva F., Herrera M.Á. (2017). The ignition index based on flammability of vegetation improves planning in the wildland-urban interface: A case study in Southern Spain. Landsc. Urban Plan..

[45] Ganteaume A. (2018). Does plant flammability differ between leaf and litter bed scale? Role of fuel characteristics and consequences for flammability assessment. Int. J. Wildl. Fire.

[46] Blackhall M., Raffaele E. (2019). Flammability of Patagonian invaders and natives: When exotic plant species affect live fine fuel ignitability in wildland-urban interfaces. Landsc. Urban Plan..

[47] Massari G., Leopaldi A. (2013). Leaf flammability in mediterranean species. Plant Biosyst..

[48] Prior L.D., Murphy B.P., Bowman D.M.J.S. (2018). Conceptualizing ecological flammability: An experimental test of three frameworks using various types and loads of surface fuels. Fire.

[49] Schwilk D.W. (2015). Dimensions of plant flammability. New Phytol..

[50] Pausas J.G., Keeley J.E., Schwilk D.W. (2017). Flammability as an ecological and evolutionary driver. J. Ecol..

[51] Ramos M.P. (2010). Manejo del Fuego.

[52] Elvira M., Luis M. (1989). Inflamabilidad y Energía de las Especies de Sotobosque. (Estudio Piloto con Aplicación a los Incendios Forestales).

[53] Morgan Varner J., Kane J.M., Kreye J.K., Engber E. (2015). The flammability of forest and woodland litter: A synthesis. Curr. For. Rep..

[54] Nuňez-Regueira L., Rodríguez-Aňón J.A., Proupín J., Mouriňo B., Artiaga-Diaz R. (2005). Energetic study of residual forest biomass using calorimetry and thermal analysis. J. Therm. Anal. Calorim..

[55] Owens M.K., Lin C.D., Taylor C.A., Whisenant S.G. (1998). Seasonal patterns of plant flammability and monoterpenoid content in Juniperus ashei. J. Chem. Ecol..

[56] Page S.E., Siegert F., Rieley J.O., Boehm H.D.V., Jaya A., Limin S. (2002). The amount of carbon released from peat and forest fires in Indonesia during 1997. Nature.

[57] Pausas J.G., Alessio G.A., Moreira B., Segarra-Moragues J.G. (2016). Secondary compounds enhance flammability in a Mediterranean plant. Oecologia.

[58] Bettinger P. (2010). An overview of methods for incorporating wildfires into forest planning models. Math. Comput. For. Nat. Resour. Sci..

[59] Castillo M.E., Molina J.R., Rodríguez y Silva F., García-Chevesich P., Garfias R. (2017). A system to evaluate fire impacts from simulated fire behavior in Mediterranean areas of Central Chile. Sci. Total Environ..

[60] Fernández I., Morales N., Olivares L., Salvatierra J., Gómez M., Montenegro G. (2010). Restauración ecológica para ecosistemas nativos afectados por incendios forestales. Rev. Chil. Hist. Nat..

[61] Miguel C.S., Roberto G.S., Guillermo J.A., Luis G.R. (2012). Análisis de grandes incendios forestales en la vegetación nativa de Chile. Interciencia.

[62] Soto M.E.C., Molina-Martínez J.R., Rodríguez y Silva F., Alvear G.H.J. (2013). A territorial fire vulnerability model for Mediterranean ecosystems in South America. Ecol. Inform..

[63] Castillo M.S., Plaza V.Á., Garfias S.R. (2020). A recent review of fire behavior and fire effects on native vegetation in Central Chile. Glob. Ecol. Conserv..

[64] CONAF Índice de Probabilidad de Ignición. www.conaf.cl/incendios-forestales/combate-de-incendios-forestales/indice-de-probabilidad-de-ignicion/.

[65] Hauenstein E., Muñoz-Pedreros A., Yánez J., Sánchez P., Möller P., Guiñez B., Gil C. (2009). Flora y vegetación de la Reserva Nacional Lago Peñuelas, Reserva de la Biósfera, Región de Valparaíso, Chile. Bosque.

[66] Dirección General De Aeronáutica Civil. Oficina de Servicios Climáticos. https://climatologia.meteochile.gob.cl/.

[67] Pickett B.M., Isackson C., Wunder R., Fletcher T.H., Butler B.W., Weise D.R. (2009). Flame interactions and burning characteristics of two live leaf samples. Int. J. Wildl. Fire.

[68] McCaw W.L., Neal J.E., Smith R.H. (1996). Fuel accumulation following prescribed burning in young even-aged stands of karri (eucalyptus diversicolor). Aust. For..

[69] Krix D.W., Murray B.R. (2018). Landscape variation in plant leaf flammability is driven by leaf traits responding to environmental gradients. Ecosphere.

[70] Krix D.W., Phillips M.L., Murray B.R. (2019). Relationships among leaf flammability attributes and identifying low-leaf-flammability species at the wildland-urban interface. Int. J. Wildl. Fire.

[71] Azcón J., Fleck I., Aranda X., Gómez N. (2008). Fotosintesis, factores ambientales. Fundam. Fisologia Veg..

[72] Restrepo H., Volder A., Lombardini L. (2009). Caracterización morfológica y fisiológica de hojas de luz y de sombra en nogal pacanero. J. Am. Soc. Hortic. Sci..

[73] Ormeño E., Ruffault J., Gutigny C., Madrigal J., Guijarro M., Hernando C., Ballini C. (2020). Increasing cuticular wax concentrations in a drier climate promote litter flammability. For. Ecol. Manag..

[74] Ganteaume A., Jappiot M., Lampin C., Guijarro M., Hernando C. (2013). Flammability of some ornamental species in wildland-urban interfaces in southeastern France: Laboratory assessment at particle level. Environ. Manag..

[75] ASTM (2000). ASTM D240-00 Standard Test Method for Heat of Combustion of Liquid Hydrocarbon Fuels by Bomb Calorimeter.

[76] ASTM (1972). ASTM D-92-72 Standard Test Method for Flash and Fire Points by Cleveland Open Cup.

[77] Ormeño E., Goldstein A., Niinemets Ü. (2011). Extracting and trapping biogenic volatile organic compounds stored in plant species. TrAC Trends Anal. Chem..

[78] Wu C., Wang F., Liu J., Zou Y., Chen X. (2015). A comparison of volatile fractions obtained from Lonicera macranthoides via different extraction processes: Ultrasound, microwave, Soxhlet extraction, hydrodistillation, and cold maceration. Integr. Med. Res..

[79] Gonçalves D., Koshima C.C., Nakamoto K.T., Umeda T.K., Aracava K.K., Gonçalves C.B., Rodrigues C.E.D.C. (2014). Deterpenation of eucalyptus essential oil by liquid + liquid extraction: Phase equilibrium and physical properties for model systems at T = 298.2 K. J. Chem. Thermodyn..

[80] Jiang J. (2005). Volatile composition of the laksa plant (*Polygonum hydropiper* L.), a potential source of green note aroma compounds. Flavour Fragr. J..

[81] Van Den Dool H., Kratz P.D. (1963). A generalization of the retention index system including linear temperature programmed gas—liquid partition chromatography. J. Chromatogr. A.

[82] Peter G. (1996). Waterman Identification of essential oil components by gas chromatography. Biochem. Syst. Ecol..

[83] Behm A.L., Duryea M.L., Long A.J., Zipperer W.C. (2004). Flammability of native understory species in pine flatwood and hardwood hammock ecosystems and implications for the wildland-urban interface. Int. J. Wildl. Fire.

[84] Bianchi L.O., Oddi F.J., Muñoz M., Defossé G.E. (2019). Comparison of leaf moisture content and ignition characteristics among native species and exotic conifers in Northwestern Patagonia, Argentina. For. Sci..

[85] Romero B., Fernandez C., Lecareux C., Ormeño E., Ganteaume A. (2019). How terpene content affects fuel flammability of wildland-urban interface vegetation. Int. J. Wildl. Fire.

[86] Programa de las Naciones Unidas para el Desarrollo (PNUD) Objetivos de Desarrollo Sostenible (ODS). www1.undp.org/content/undp/es/home/sustainable-development-goals.html..

[87] Ganteaume A., Guijarro M., Jappiot M., Hernando C., Lampin-Maillet C., Pérez-Gorostiaga P., Vega J.A. (2011). Laboratory characterization of firebrands involved in spot fires. Ann. For. Sci..

[88] Nunes A.N., Lourenço L., Meira A.C.C. (2016). Exploring spatial patterns and drivers of forest fires in Portugal (1980–2014). Sci. Total Environ..

[89] Rodríguez Rivas A. (2009). Estudios de Valoración Energética de Combustibles Forestales para la Prevención de Incendios Forestales en la Sierra de la Primavera (Jalisco, México) Mediante Calorimetría de Combustión y Ensayos de Inflamabilidad.

[90] Ormeño E., Fernandez C., Mévy J.P. (2007). Plant coexistence alters terpene emission and content of Mediterranean species. Phytochemistry.

[91] Dimitrakopoulos A.P., Papaioannou K.K. (2001). Flammability Assessment of Mediterranean Forest Fuels. Fire Tech..

[92] Vecchio M.G., Loganes C., Minto C. (2016). Beneficial and Healthy Properties of Eucalyptus Plants: A Great Potential Use. Open Agric. J..

[93] Nowak D.J., Crane D.E., Stevens J.C., Ibarra M. (2002). Brooklyn’s Urban Forest.

[94] Di Cosmo D., Santander R., Urzúa A., Palacios S.M., Rossi Y. (2015). Insecticidal effect of Cryptocarya alba essential oil on the housefly, Musca domestica L. Bol. Latinoam. Caribe Plantas Med. Aromat..

[95] Bravo J., Carbonell V., Sepúlveda B., Delporte C., Valdovinos C.E., Martín-Hernández R., Higes M. (2017). Antifungal activity of the essential oil obtained from Cryptocarya alba against infection in honey bees by Nosema ceranae. J. Invertebr. Pathol..

[96] Murray B.R., Hardstaff L.K., Phillips M.L. (2013). Differences in leaf flammability, leaf traits and flammability-trait relationships between native and exotic plant species of dry sclerophyll forest. PLoS ONE.

[97] De Lillis M., Bianco P.M., Loreto F. (2009). The influence of leaf water content and isoprenoids on flammability of some Mediterranean woody species. Int. J. Wildl. Fire.

[98] Dhifi W., Bellili S., Jazi S., Bahloul N., Mnif W. (2016). Essential Oils’ Chemical Characterization and Investigation of Some Biological Activities : A critical Review. Medicines.

[99] Grootemaat S., Wright I.J., van Bodegom P.M., Cornelissen J.H.C., Cornwell W.K. (2015). Burn or rot: Leaf traits explain why flammability and decomposability are decoupled across species. Funct. Ecol..

[100] Jolly W.M., Parsons R.A., Hadlow A.M., Cohn G.M., McAllister S.S., Popp J.B., Hubbard R.M., Negron J.F. (2012). Relationships between moisture, chemistry, and ignition of Pinus contorta needles during the early stages of mountain pine beetle attack. For. Ecol. Manag..

[101] Qi Y., Jolly W.M., Dennison P.E., Kropp R.C. (2016). Seasonal relationships between foliar moisture content, heat content and biochemistry of lodgepole line and big sagebrush foliage. Int. J. Wildl. Fire.

