# Supplementary material for: Leaf Thermal and Chemical Properties as Natural Drivers of Plant Flammability of Native and Exotic Tree Species of the Valparaíso Region, Chile
Source: Int J Environ Res Public Health. 2021 Jul 5;18(13):7191. doi: 10.3390/ijerph18137191 (PMC8297029; doi:10.3390/ijerph18137191)
Supplement: Supplementary file 1 [file ijerph-18-07191-s001.zip › ijerph-1229485-supplementary.pdf]

Supplementary Materials

Leaf thermal and chemical properties as natural drivers of plant flammability of native and exotic tree species of the Valparaíso region, Chile

Supplementary Tables

Table S1. Multiple comparisons using the HSD Tukey test of flammability parameters of ignition time (IT), flame duration (FD), and total burning time (BT).

Variable	Species	Species	Mean differences	Deviation	Significance	Lower limit	Upper limit
IT	*Q. saponaria*	*C. alba*	14.13*	0.65	0.00	12.33	15.93
	Q. saponaria	*E. globulus*	17.01*	0.65	0.00	15.21	18.81
	Q. saponaria	*P. radiata*	1.45	0.65	0.18	−0.35	3.25
	Q. saponaria	*A. dealbata*	11.34*	0.65	0.00	9.54	13.14
	C. alba	*E. globulus*	2.87*	0.65	0.00	1.07	4.67
	C. alba	*P. radiata*	−12.68*	0.65	0.00	−14.48	−10.88
	C. alba	*A. dealbata*	−2.79*	0.65	0.00	−4.59	−0.99
	E. globulus	*P. radiata*	−15.55*	0.65	0.00	−17.35	−13.75
	E. globulus	*A. dealbata*	−5.66*	0.65	0.00	−7.46	−3.86
	P. radiata	*A. dealbata*	9.88*	0.65	0.00	8.08	11.68
FD	*Q. saponaria*	*C. alba*	2.76*	0.90	0.02	0.28	5.23
	Q. saponaria	*E. globulus*	1.97	0.90	0.18	−0.50	4.44
	Q. saponaria	*P. radiata*	1.39	0.900	0.53	−1.07	3.87
	Q. saponaria	*A. dealbata*	−6.64*	0.90	0.00	−9.11	−4.17
	C. alba	*E. globulus*	−0.79	0.90	0.90	−3.26	1.68
	C. alba	*P. radiata*	−1.36	0.90	0.55	−3.84	1.10
	C. alba	*A. dealbata*	−9.40*	0.90	0.00	−11.88	−6.93
	E. globulus	*P. radiata*	−0.57	0.900	0.96	−3.04	1.90
	E. globulus	*A. dealbata*	−8.61*	0.900	0.00	−11.08	−6.14
	P. radiata	*A. dealbata*	−8.04*	0.900	0.00	−10.51	−5.56
BT	*Q. saponaria*	*C. alba*	14.47*	1.24	0.00	11.05	17.88
	Q. saponaria	*E. globulus*	8.90*	1.24	0.00	5.48	12.31
	Q. saponaria	*P. radiata*	13.98*	1.24	0.00	10.56	17.39
	Q. saponaria	*A. dealbata*	12.04*	1.24	0.00	8.63	15.45
	C. alba	*E. globulus*	−5.57*	1.24	0.00	−8.98	−2.15
	C. alba	*P. radiata*	−0.49	1.24	0.99	−3.90	2.92
	C. alba	*A. dealbata*	−2.42	1.24	0.29	−5.83	0.98
	E. globulus	*P. radiata*	5.08*	1.24	0.01	1.66	8.49
	E. globulus	*A. dealbata*	3.14	1.24	0.08	−0.26	6.55
	P. radiata	*A. dealbata*	−1.93	1.24	0.52	−5.34	1.47

* Present a statistically significant difference. No star would indicate no statistically significant difference. .

Table S2. Multiple range test through the percent LSD/HSD test with the confidence interval at 95% of higher heating value (HHV), lower heating value (LHV), flash point (FP), and moisture content (MC).

Variable	Species	Species	Significance	Difference	+/- Limits
HHV	*A. dealbata*	*E. globulus*	*	−0.59	0.29
	A. dealbata	*C. alba*	*	0.93	0.29
	A. dealbata	*P. radiata*	*	0.99	0.29
	A. dealbata	*Q. saponaria*	*	2.38	0.29
	E. globulus	*C. alba*	*	1.53	0.29
	E. globulus	*P. radiata*	*	1.59	0.29
	E. globulus	*Q. saponaria*	*	2.98	0.29
	C. alba	*P. radiata*		0.06	0.29
	C. alba	*Q. saponaria*	*	1.44	0.29
	P. radiata	*Q. saponaria*	*	1.38	0.29

**LHV**	*A. dealbata*	*E. globulus*	*	−0.74	0.28
	A. dealbata	*C. alba*	*	1.94	0.28
	A. dealbata	*P. radiata*	*	−1.03	0.28
	A. dealbata	*Q. saponaria*	*	3.06	0.28
	E. globulus	*C. alba*	*	1.20	0.28
	E. globulus	*P. radiata*	*	0.29	0.28
	E. globulus	*Q. saponaria*	*	2.31	0.28
	C. alba	*P. radiata*	*	0.91	0.28
	C. alba	*Q. saponaria*	*	−1.11	0.28
	P. radiata	*Q. saponaria*	*	2.02	0.28
**FP**	*A. dealbata*	*E. globulus*		3.5	68.68
	A. dealbata	*C. alba*		15.0	68.68
	A. dealbata	*P. radiata*		−17.5	68.68
	A. dealbata	*Q. saponaria*		−35.0	68.68
	E. globulus	*C. alba*		11.5	68.68
	E. globulus	*P. radiata*		−21.0	68.68
	E. globulus	*Q. saponaria*		−38.5	68.68
	C. alba	*P. radiata*		−32.5	68.68
	C. alba	*Q. saponaria*		−50.0	68.68
	P. radiata	*Q. saponaria*		−17.5	68.68
**MC**	*A. dealbata*	*E. globulus*		5.0	9.19
	A. dealbata	*C. alba*	*	56.5	9.19
	A. dealbata	*P. radiata*	*	46.0	9.19
	A. dealbata	*Q. saponaria*	*	14.0	9.19
	E. globulus	*C. alba*	*	51.5	9.19
	E. globulus	*P. radiata*	*	41.0	9.19
	E. globulus	*Q. saponaria*		9.0	9.19
	C. alba	*P. radiata*	*	−10.5	9.19
	C. alba	*Q. saponaria*	*	−42.5	9.19
	P. radiata	*Q. saponaria*	*	−32.0	9.19

* Present a statistically significant difference. No star would indicate no statistically significant difference.

Table S3. Identification of organic compounds by chromatography (GC/MS) contained in the essential oils (EOs) of exotic and native species in summer 2018–2019.

						Exotic species			Native species	
						EG	PR	AD	CA	QS
**RI**	**RT**	**Compound**	**Molecular weight (g mol⁻¹)**	**CC**	**Flash point (°C)**	**Relative content**		**(%)**	**Relative content (%)**	
958	8.57	octano, 4 etil	-	AH	-	ND	0.60	2.16	0.12	ND
1199	13.22	Dodecane	170.33	AH	71.1	ND	0.52	ND	0.08	ND
1386	15.93	Unknown sesquiterpene	-	ST	-	0.79	ND	ND	ND	ND
1389	15.97	α-Ylangene	204.35	ST	105.1	ND	ND	ND	0.16	ND
1403	16.16	β-Elemene	204.35	ST	98.3	ND	ND	ND	0.23	ND
1426	16.46	α-Gurjunene	204.35	ST	101.3	2.77	ND	ND	ND	ND
1438	18.59	(E)-β-Caryophyllene	204.35	ST	104.9	1.22	6.34	ND	ND	ND
1445	16.71	Patchoulene	204.35	ST	110.2	1.03	ND	ND	ND	ND
1446	16.72	α-Bergamotene	204.35	ST	100.6	ND	1.64	ND	1.97	ND
1451	16.78	β-Copaene	204.35	ST	104.9	5.77	ND	ND	ND	ND
1457	16.86	Aromadendrene	204.35	ST	106.5	19.26	ND	ND	ND	0.44
1461	19.02	(E)-β-Farnesene	204.35	ST	109.7	ND	ND	ND	0.39	ND
1472	17.05	cis-α-Bisabolene	204.35	ST	110.5	1.36	1.12	ND	0.23	ND
1480	17.15	Alloaromadendrene	204.35	ST	106.5	47.37	0.70	ND	0.22	1.65
1491	17.21	Unknown sesquiterpene	-	ST	-	1.27	0.40	ND	0.15	ND
1498	17.40	Germacrene D	204.35	ST	111.9	ND	4.05	ND	1.78	ND

1505	17.47	Unknown sesquiterpene	-	ST	-	1.71	ND	ND	0.19	ND
1508	17.51	Unknown sesquiterpene	-	ST	-	1.03	ND	ND	ND	ND
1513	19.61	α-Farnesene	204.35	ST	113.2	ND	1.45	ND	ND	ND
1514	17.57	Unknown sesquiterpene	-	ST	-	ND	ND	ND	0.20	ND
1515	17.60	α-Chamigrene	204.35	ST	107.0	1.56	0.96	ND	ND	ND
1517	19.90	Unknown sesquiterpene	-	ST	-	ND	ND	ND	0.19	ND
1526	17.72	Lauric acid. methyl ester	214.34	ES	114.6	ND	27.75	ND	ND	ND
1531	17.79	Unknown sesquiterpene	-	ST	-	1.02	ND	ND	0.19	ND
1538	17.87	Unknown sesquiterpene	-	ST	-	ND	1.05	ND	ND	ND
1539	20.20	trans-Calamene	202.34	ST	125.4	ND	ND	ND	0.35	ND
1540	17.89	Unknown sesquiterpene	-	ST		1.19	ND	ND	ND	ND
1600	18.62	Hexadecane	226.44	AH	135.0	ND	ND	ND	ND	0.86
1618	18.83	Unknown sesquiterpene	-	ST	-	ND	ND	ND	0.21	ND
1633	19.00	Unknown sesquiterpene	-	ST	-	ND	ND	ND	0.21	ND
1726	20.05	Methyl tetradecanoate	242.40	ES	134.6	ND	7.11	ND	ND	0.80
1799	20.85	Octadecane	254.49	AH	165.6	ND	ND	ND	ND	1.05
1841	21.28	3.7.11.15-tetramethyl-2-hexadecen-1-ol	296.53	OH	-	ND	ND	ND	ND	1.29
1928	22.15	Palmitic acid. methyl ester	270.45	ES	152.8	3.67	4.61	4.61	4.28	12.02
2000	22.86	Eicosane	282.55	AH	186.5	ND	ND	ND	ND	0.61
2099	23.80	Linoleic acid. methyl ester	294.47	ES	96.9	ND	ND	ND	1.67	ND
2100	23.81	Heneicosane	296.57	AH	198.8	ND	ND	ND	ND	7.69
2101	23.81	Methyl linoleate	294.47	ES	96.9	ND	1.98	ND	ND	ND
2109	23.88	Methyl linolenate	292.46	ES	101.4	ND	9.00	ND	6.00	17.79
2129	24.07	Methyl stearate	298.50	ES	169.3	ND	ND	1.70	ND	1.10
2198	24.70	Docosane	310.60	AH	210.9	ND	0.40	ND	ND	0.35
2236	25.02	Andrographolide	350.45	DT	195.5	ND	1.26	ND	ND	ND
2332	25.83	Methyl 18-methylnonadecanoate	326.56	ES	-	ND	2.40	ND	ND	ND
2400	26.40	Tetracosane	338.65	AH	234.5	ND	ND	ND	0.43	0.47
2498	27.19	Pentacosane	352.68	AH	246.1	ND	ND	ND	0.31	ND
2531	27.43	Unknown hydrocarbon	-	-	-	ND	ND	ND	0.29	ND
2533	27.45	Docosanoic acid. methyl ester	354.61	ES	197.7	ND	4.44	ND	ND	ND
2597	27.94	Hexacosane	366.71	AH	257.6	ND	ND	ND	0.69	0.98
2699	28.70	Heptacosane	380.70	AH	268.9	3.90	ND	ND	7.32	ND
2799	29.41	Octacosane	394.76	AH	280.1	ND	ND	ND	1.48	0.88
2899	30.09	Nonacosane	408.79	AH	291.2	5.08	ND	78.37	8.50	31.99
3016	30.96	Unknown diterpene	-	DT	-	ND	ND	ND	1.01	ND
3045	31.20	Unknown hydrocarbon	-	-	-	ND	ND	ND	8.13	ND
3098	31.62	Hentriacontane	436.84	AH	313.1	ND	ND	ND	1.28	ND
3151	32.12	Unknown triterpene	-	TP	-	ND	12.90	ND	ND	ND
3187	32.46	Unknown hydrocarbon	-	-	-	ND	ND	ND	9.04	ND
3248	33.10	Unknown hydrocarbon	-	-	-	ND	ND	ND	3.46	ND
3292	33.59	16-Hentriacontanone	450.82	KO	35.6	ND	ND	ND	35.60	ND

RI: Retention index; RT: Retention time; ND: Not detected; CC: classification of organic compounds; EG: *E. globulus*; PR: *P. radiata*; AD: *A. dealbata*; CA: *C. alba*; QS: *Q. Saponaria*; ST: Sesquiterpene; AH: Aliphatic hydrocarbon; ES: Ester; KO: Ketone; OH: Alcohol; DT: Diterpene; TP: Triterpene.

Table S4. Simple regression analysis between parameters of flammability, heating values, and natural driver of leaves with a confidence level of 95%. EOs and MC, independent variables: x; IT, FD and BT, dependent variables: y; and heating value, HHV and LHV, dependent/independent variables: y/x.

**Parameters**	**EOs**	**MC**	**FP**	**HHV**	**LHV**
**IT**	F = 1.51; p = 0.31 R = −0.58; R^2 = 0.33	F = 0.07; p = 0.81; R = −0.15; R^2 = 0.23	F = 14.11; p = 0.03; R = 0.91; R^2 = 0.82	F = 5.74; p = 0.10; R = −0.81; R^2 =0.66	F = 0.98; p = 0.40; R = −0.50; R^2 = 0.25
**FD**	F = 1.61; p = 0.30 R = −0.59; R^2 = 0.35	F = 1.96; p = 0.26 R = 0.63; R^2 = 0.40	F = 0.01; p = 0.93 R = 0.06; R^2 = 0.34	F = 0.11; p = 0.77 R = 0.18; R^2 = 0.34	F = 1.01; p = 0.39 R = 0.50; R^2 = 0.25
**BT**	F = 1.55; p = 0.30 R = −0.58; R^2 = 0.34	F = 1.23; p = 0.35 R = 0.54; R^2 = 0.29	F = 3.85; p = 0.14 R = 0.75; R^2 = 0.56	F = 1.09; p = 0.37 R = −0.52; R^2 = 0.27	F = 1.63; p = 0.29 R = −0.59; R^2 = 0.35
**HHV**	F = 2,23; p = 0.23 R = 0.65; R^2 = 0.43	F = 0.38; p = 0.58 R = 0.34; R^2 = 0.11	F = 3.20; p = 0.17 R = −0.72; R^2 = 0.52		
**LHV**	F = 0.55; p = 0.51 R = 0.39; R^2 = 0.16	F = 0.36; p = 0.59 R = 0.33; R^2 = 0.11	F = 1.10; p = 0.37 R = −0.52; R^2 = 0.27		
**FP**	F = 1.67; p = 0.29 R = −0,60; R^2 = 0.36	F = 0.12; p = 0.76 R = 0.19; R^2 = 0.04			
